# Plant leaf veins coupling feature representation and measurement method based on DeepLabV3+

**DOI:** 10.3389/fpls.2022.1043884

**Published:** 2022-11-24

**Authors:** Xiaobao Liu, Biao Xu, Wenjuan Gu, Yanchao Yin, Hongcheng Wang

**Affiliations:** Faculty of Mechanical and Electrical Engineering, Kunming University of Science and Technology, Kunming, China

**Keywords:** plant leaf veins, coupled features, lightweight, convex hull-scan, F-3MS refinement algorithm

## Abstract

The plant leaf veins coupling feature representation and measurement method based on DeepLabV3+ is proposed to solve problems of slow segmentation, partial occlusion of leaf veins, and low measurement accuracy of leaf veins parameters. Firstly, to solve the problem of slow segmentation, the lightweight MobileNetV2 is selected as the extraction network for DeepLabV3+. On this basis, the Convex Hull-Scan method is applied to repair leaf veins. Subsequently, a refinement algorithm, Floodfill MorphologyEx Medianblur Morphological Skeleton (F-3MS), is proposed, reducing the burr phenomenon of leaf veins’ skeleton lines. Finally, leaf veins’ related parameters are measured. In this study, mean intersection over union (MIoU) and mean pixel accuracy (mPA) reach 81.50% and 92.89%, respectively, and the average segmentation speed reaches 9.81 frames per second. Furthermore, the network model parameters are compressed by 89.375%, down to 5.813M. Meanwhile, leaf veins’ length and width are measured, yielding an accuracy of 96.3642% and 96.1358%, respectively.

## 1 Introduction

The leaf veins are essential constituents of the leaves, which play a crucial role in transporting and supporting, regarded as the “transporters” of plant nutrients. In agriculture, leaf veins’ parameters are significant indicators of a crop’s growth state. Leaf veins’ length and thickness can be used to judge the ability to transport water, inorganic salts, and trace elements (Konch et al., 2021; Pan et al., 2022). After measuring the leaf veins, the obtained parameters can be used to analyze the efficiency of leaf veins in transporting nutrients, which is crucial for the morphogenesis of leaf veins (Ma et al., 2021). The morphology of leaves can also be judged through leaf veins, by which the speed of photosynthesis is predicted (Huang et al., 2022; Zhang et al., 2022). Meanwhile, leaf vein morphology is associated with specific climatic oscillations and varies within species along altitudinal gradients. By measuring the leaf veins’ parameters, the quality of the local ecosystem can be reflected (Rodríguez-Ramírez et al., 2021). Therefore, to achieve a quantitative analysis of the growth state of crops, leaf veins’ segmentation and parameters’ measurement techniques are paramount for agriculture (Li et al., 2006; Radha and Jeyalakshmi, 2014; Zhu et al., 2020). However, the state of leaf veins in crops is usually judged by farmers based on personal experience traditionally, which is time-consuming and labor-intensive. There is a certain misjudgment in the crop growth state by observing the leaf vein morphology. To some extent, it may cause losses to national agricultural production. Hence, identifying leaf veins quickly and measuring parameters accurately is an urgent demand. In this study, the problems mentioned above can be solved with the help of lightweight deep neural networks and leaf vein repair technology. Subsequently, the measurement of leaf veins’ related parameters is more accurate.

The leaf veins contain important plant physiological information (Wen et al., 2018; Ye et al., 2021; Huang et al., 2022). With the rapid development of digital image processing technology, many scholars analyzed plant leaf veins with Software Aids, Morphological Image Processing, Color Space, and traditional machine learning algorithms. For example, (Bühler et al., 2015) measured vein-related parameters with the phenoVein Image Analysis Tool based on manually corrected leaf veins, before which veins information was highlighted by compensating for the local uneven brightness. (Jiyou et al., 2019) chose eCognition software to perform multi-scale segmentation of plant leaf veins in the microscopic state, and subsequently, a leaf vein extraction data parameters library was built through the relevant spectral and geometric information, which can be applied to study the correlation mechanism of plant leaf ecology. However, repeated manual operations and the method’s low efficiency lead to no real-time segmentation. (Zheng and Wang, 2010a) proposed a morphology-based algorithm for leaf vein extraction, in which a threshold segmentation method was brought forward, using digital morphology operation based on the leaf images processed to grayscale. (Li et al., 2018) proposed a method to detect leaf veins by combining fuzzy logic with sequential morphology. The Sugeno blur model, which could enhance contrast for leaf vein images, was combined with a composite sequential morphological detection algorithm to detect leaf veins. (Zheng and Wang, 2010b) presented a grayscale morphology method to extract leaf veins. In this research, the morphology was selected to eliminate overlap color between leaf veins and the background in a grayscale image, and the color difference was adjusted through linear intensity. Finally, the OUST method was used to separate leaf veins from the background. However, morphological processing can only be applied to simple leaves, and large non-leaf vein regions were extracted for more complex leaves. (Li et al., 2011) combined hue information with an improved Sobel operator to extract leaf veins by transferring the leaves’ image into HIS color space. Nevertheless, due to differences in leaf color for each crop, after transitioning to a different color space, there were still significant errors in the human senses to adjust each color space, which cannot perform batch extraction. Meanwhile, a multitude of traditional machine learning algorithms were proposed to extract leaf veins. (Selda et al., 2017) introduced a method for plant identification through leaf vein images, in which SVM was used to classify plant species based on the features detected by the SIFT algorithm. (Lee and Hong, 2013) developed an identification system of plant leaves through leaf veins and shape. The core idea is to use Fast Fourier Transform (FFT) method to identify leaf vein features. (Samanta et al., 2021) identified plant categories through three tree leaf vein parameters (jackfruit, mango, and linden) based on the K-means clustering and K-Nearest Neighbors (KNN) technique. Nevertheless, some traditional machine learning algorithms do not accept missing data. For instance, SVM is very sensitive to outliers in the datasets. K-means and KNN require the pre-input of cluster classes, leading to unadaptable feature extraction. Therefore, these algorithms cannot be well applied in some complex agricultural fields.

In recent years, convolutional neural networks (CNNs) have been used for many advanced computer vision tasks (Westphal and Seitz, 2021; Ulku and Akagündüz, 2022; Lu et al., 2022a). Meanwhile, the advantages of CNN-based autonomous feature extraction have a wide range of applications for agriculture (Ghazi et al., 2017; Barré et al., 2017; Too et al., 2019). Additionally, image segmentation tasks are also widely put into use in crop recognition. (Deepalakshmi et al., 2021) used the CNN algorithm to extract features from the input image to distinguish between healthy and diseased leaves. (Beikmohammadi et al., 2022) proposed a novel multi-stage method to identify leaves based on deep CNN. This study uses the computer vision task of leaf classification to recognize plant species automatically. The apple leaf segmentation method was introduced based on an asymmetric mixed-wash convolutional neural network (Zifen et al., 2021). In this research, by using the asymmetric shuffling module to replace the original convolution module, the receptive field was improved for higher segmentation accuracy. (Fuentes-Pacheco et al., 2019) introduced a plant segmentation network based on an encoder-decoder framework. In this study, the onboard camera was used to photograph crops, and the CNN was used to learn the difference in the leaf’s appearance to achieve pixel segmentation of crops and non-crops. (Lu et al., 2022b) proposed a plant leaf segmentation and feature extraction method based on multi-view time series images, which was achieved through segmenting stems and leaves of arabidopsis, corn, and physalis by Mask-RCNN. (Bosilj et al., 2020) used transfer learning to reduce the retraining time and labeling effort required for new crops, in which crops and weeds were segmented in precision agriculture. (Milioto et al., 2018) proposed real-time semantic segmentation of crops and weeds using precision agriculture robots by leveraging background knowledge in CNNs. In this study, the training weights achieved by neural networks were implanted into precision robots to remove weeds from field sugar beets. Many scholars have studied plant leaves through semantic segmentation (Barth et al., 2018; Miao et al., 2020; Kolhar and Jagtap, 2021; Masuda, 2021). However, the conventional semantic segmentation network has a large number of parameters and needs a long training time. Moreover, the segmentation is less effective with a small proportion of target pixels, which cannot meet the accuracy requirements of real-time segmentation in agriculture.

This study aims to develop a method of real-time segmentation and parameter measurement for plant leaf veins that can be applied in agriculture to solve problems of slow segmentation and large parameter measurement errors. Specifically, the DeepLabV3+ semantic segmentation network is selected as the basic framework (Chen et al., 2018). The main contributions of this study are as follows:

1. The Xception feature extraction network is replaced with the lightweight MobileNetV2 (Sandler et al., 2018). It greatly reduces the number of model parameters, shortens the network’s training time, and meets the requirements of online real-time segmentation of leaf veins.
2. A Convex Hull-Scan method is proposed to repair and obtain more complete leaf veins, improving the measurement accuracy of leaf veins’ parameters.
3. In repaired leaf veins, there are still fine cavities and slightly rough veins’ contours, resulting in a large number of burrs when extracting leaf veins’ skeleton lines, which affects the accuracy of the parameter measurement. An F-3MS refinement algorithm is adopted to reduce the burr phenomenon.

The remainder of the study is organized as follows: In Section 2, the acquisition of image datasets is described in detail. The MoileNetV2-DeepLabV3+ lightweight network is constructed to segment leaf veins. This study developed two methods, the Convex Hull-Scan method and the F-3MS refinement algorithm, which can repair leaf veins incompletely segmented and eliminate burrs along leaf veins’ skeleton lines. The length of the leaf veins is also measured. Finally, a width geometric model is constructed to measure the leaf vein width. In Section 3, the implementation details of the experiment are presented. In section 4, a detailed experimental analysis is carried out to verify the algorithm’s feasibility. Section 5 introduced the algorithm’s benefits, limitations, and follow-up work. Section 6 concludes this study and provides an outlook for the future.

## 2 Materials and methods

The data used for this research, including data acquisition, split, and annotation, is presented in Section 2.1. The model construction is introduced in Section 2.2. Measuring leaf vein parameters is elaborated in detail in Section 2.3.

### 2.1 Data

#### 2.1.1 Data acquisition

**Figure 1:**
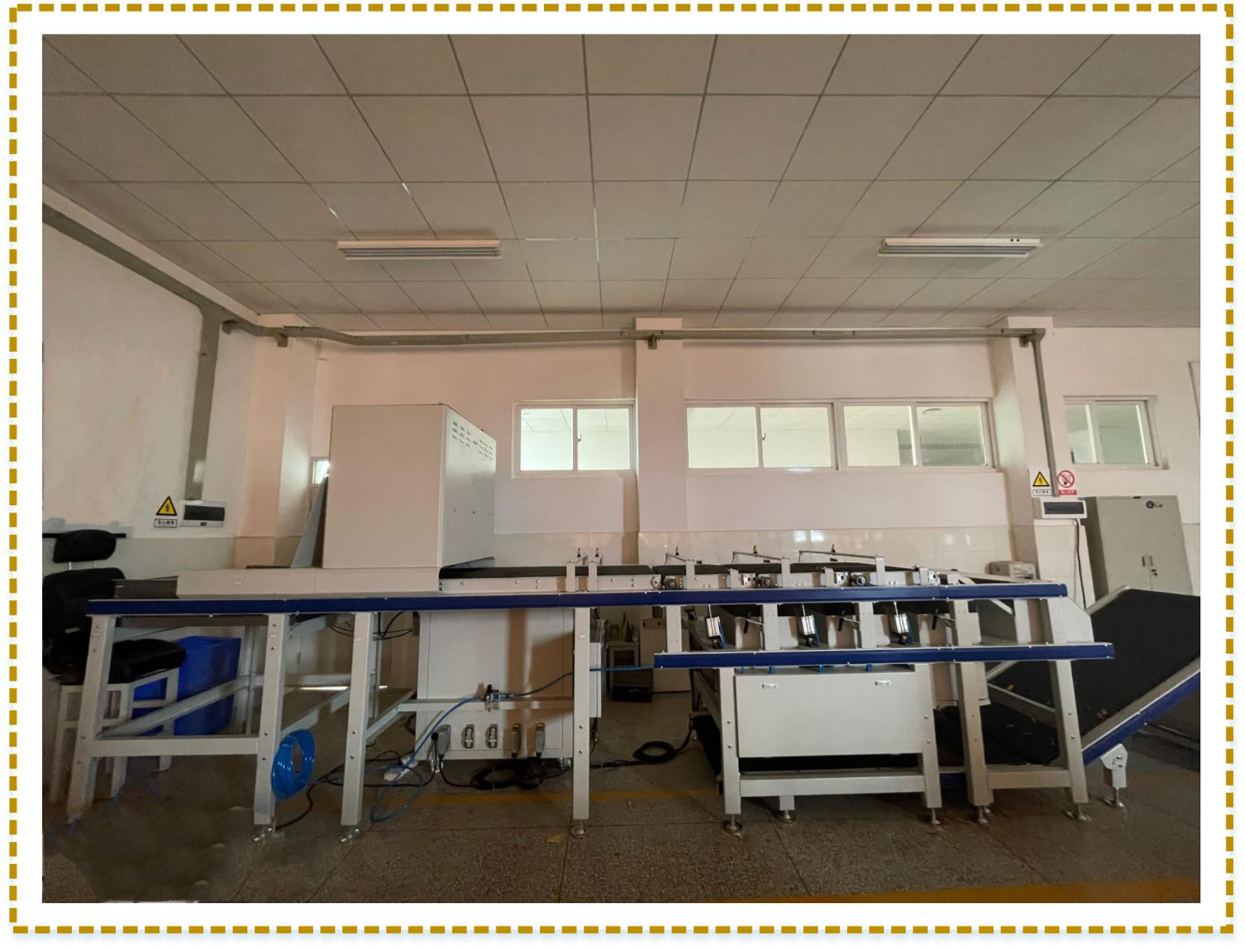
Professional acquisition machine for images. The image data collector consists of conveyor belts, industrial cameras, operating systems, sensors, collecting boxes, etc.

The experimental data are obtained from Yunnan Tobacco Quality Supervision and Monitoring Station in Kunming, China, and the image data are flue-cured tobacco leaves. The image acquisition platform is provided by an agricultural company. During image data acquisition, the equipment used is a Hikvision MV-CA050-11U camera with a USB interface. The light source model is XC-BK-650-1100, the lens model is M0824-MPW2, and the light source is customized to 1000×600. The shooting distance between the lens and the leaf surface is 90 cm. These images were collected in November 2021. The flue-cured tobacco leaves were placed on the conveyor belt. This study uses RGB images with a resolution of 2304×1520 pixels. A total of 800 images were acquired. The detailed equipment for data acquisition is shown in **Figure 1**.

#### 2.1.2 Data annotation

**Figure 2:**
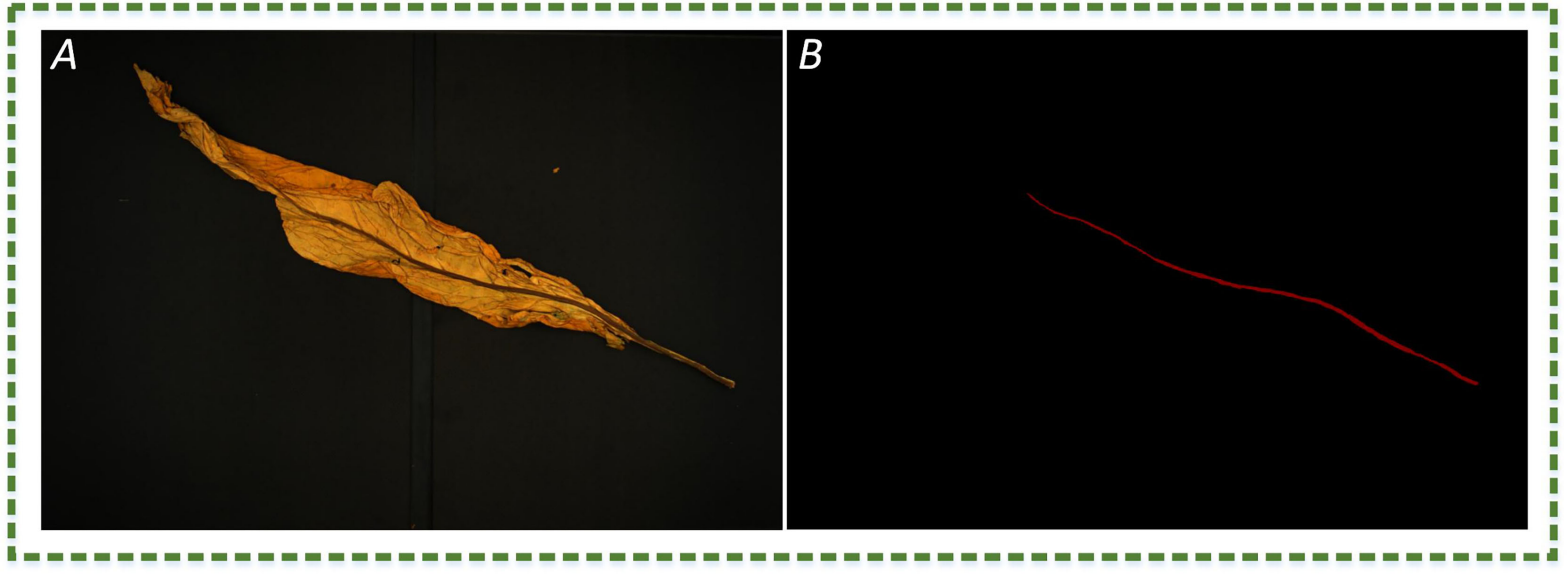
The image example. **(A)** The RGB image of plant leaves; **(B)** manual annotation of leaf vein region, where red and black represent the leaf vein and background, respectively.

This study uses supervised learning for neural network training. Therefore, the region of leaf veins should be annotated so that the network can recognize them. Each pixel on the images is annotated as a leaf vein and background class by using the open-source annotation program Labelme3.16.7. A visual example is shown in **Figure 2**. It is worth noting that leaf veins are thin strips in shape, and per pixel-level annotation is very time-consuming. The annotation mask work was accomplished in almost two months.

#### 2.1.3 Data split

This study mainly focuses on the analysis of plants’ main veins. Scaling, translation, mirroring, and rotation are used in image data augmentation for enhancing generalization ability, improving segmentation accuracy, and preventing overfitting. These augmented RGB images are divided into training, validation, and test sets (at 8:1:1 ratio). Finally, the datasets are made according to the PASCAL VOC.

### 2.2 Model construction

This section illustrates the network construction in detail. MobileNetV2-DeepLabV3+ is introduced in Section 2.2.1. The lightweight extraction network is elaborated in Section 2.2.2. And Section 2.2.3 presents a Convex Hull-Scan method.

**Figure 3:**
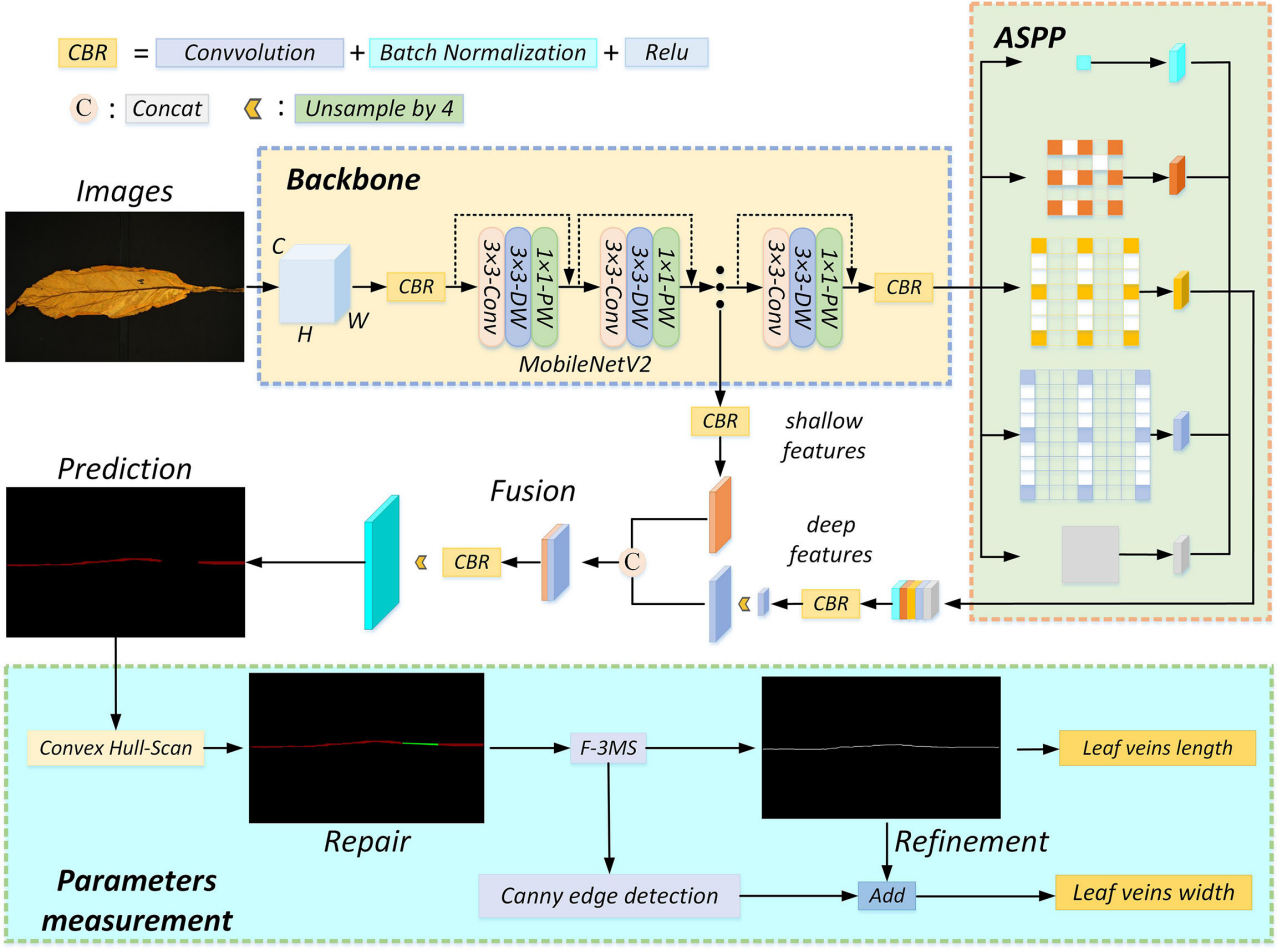
The algorithm flow chart. The inputted RGB image goes through four processes: feature extraction, occlusion region repair, leaf vein skeleton line extraction, and leaf vein parameter measurement. CBR consists of convolution, batch normalization, and activation function layers. Fusion means “Concat” splicing shallow features with deep features. “Unsample by 4” means that the feature map is upsampled by 4, and the model uses 2 upsampling operations.

In this study, the plant leaf veins coupling feature representation and measurement method based on DeepLabV3+ is proposed to solve problems of slow segmentation, partial occlusion of leaf veins, and low measurement accuracy of leaf veins parameters. The flowchart involved in leaf vein segmentation and parameter measurement is shown in **Figure 3**. The RGB image is uniformly scaled to 512 × 512 pixels and input into the network. The MobileNetV2-DeepLabV3+ is used to segment leaf veins. The Convex Hull-Scan method is used to repair and form a complete vein, and the F-3MS algorithm removes the blur phenomenon of the vein’s skeleton lines.

#### 2.2.1 MobileNetV2-DeepLabV3+ network

Semantic segmentation networks can meet most conventional objects’ accuracy of segmentation and recognition. However, the segmentation accuracy for objects with small pixel proportions is still low. Moreover, limited computer resources are far from meeting the purpose of real-time segmentation in practical application scenarios. To solve the problem of slow segmentation, MobileNetV2 is chosen as the feature extraction network for DeepLabV3+. Firstly, the image (512×512 pixels, 3 channels) is inputted to generate a feature map (32×32 pixels, 320 channels) by the MobileNetV2. The feature map (32×32 pixels, 320 channels) is passed into atrous spatial pyramid pooling (ASPP) to obtain deeper semantic information, followed by upsampling and concatenation with the shallow feature map (128×128 pixels, 24 channels). Finally, the concatenated feature map is convolved and up-sampled to obtain leaf veins. The MobileNetV2-DeepLabV3+ can ensure the needed accuracy while improving the segmentation speed.

#### 2.2.2 Lightweight feature extraction network

The residual structure of the Xception module is used for feature extraction by three times 3 × 3 depthwise separable convolutions in the original DeepLabV3+. As the number of channels in a convolutional layer is up to 512, the network runs slowly due to the computer resources occupied by a large number of model parameters. To solve this problem without sacrificing accuracy, the lightweight network used can improve the training speed to achieve real-time segmentation (Ma et al., 2018; Zhang et al., 2018; Chaturvedi et al., 2020; Wang et al., 2020).

Common feature representation methods include the Local Binary Patterns (LBP) algorithm (Zhao and Pietikainen, 2007), the Histogram of Oriented Gradient (HOG) feature extraction algorithm (Dalal and Triggs, 2005), and the Scale-invariant feature transform (SIFT) operator (Cheung and Hamarneh, 2009). In computer vision tasks, image features are mainly composed of color, geometry, texture, and local features. A single feature representation method for complex image segmentation tasks cannot obtain the required target. The convolutional neural network based on deep learning can automatically learn and obtain feature representation. The features learned by the shallow layer are simple edges, corners, textures, geometric shapes, surfaces, etc., and the features learned by the deep layer are more complex and abstract. When representing images, the feature values might correspond to the pixels of an image. The convolution kernel weights of each convolutional neural network layer are learned by data-driven learning. Data-driven convolutional neural networks learn features from simple to complex layer by layer, and complex patterns are composed of simple patterns. This combination is carried out relatively flexibly, so it has a solid ability to obtain feature representation.

The regions of leaf veins occupy a small proportion of pixels in the entire image. Continuous convolution and pooling operations lead to the loss of leaf veins’ feature information when the feature extraction network layers are too deep. Meanwhile, the deep network layers lead to a large number of model parameters and a long training time. The feature extraction network with shallow network layers only extracts the color and texture information of the leaf veins, and the deeper semantic information cannot be extracted. Hence, MobileNetV2 is used for plant leaf veins’ extraction in this study. While ensuring that the network layer is deep enough, many depthwise separable convolutions are used to reduce model parameters and shorten training time.

**Figure 4:**
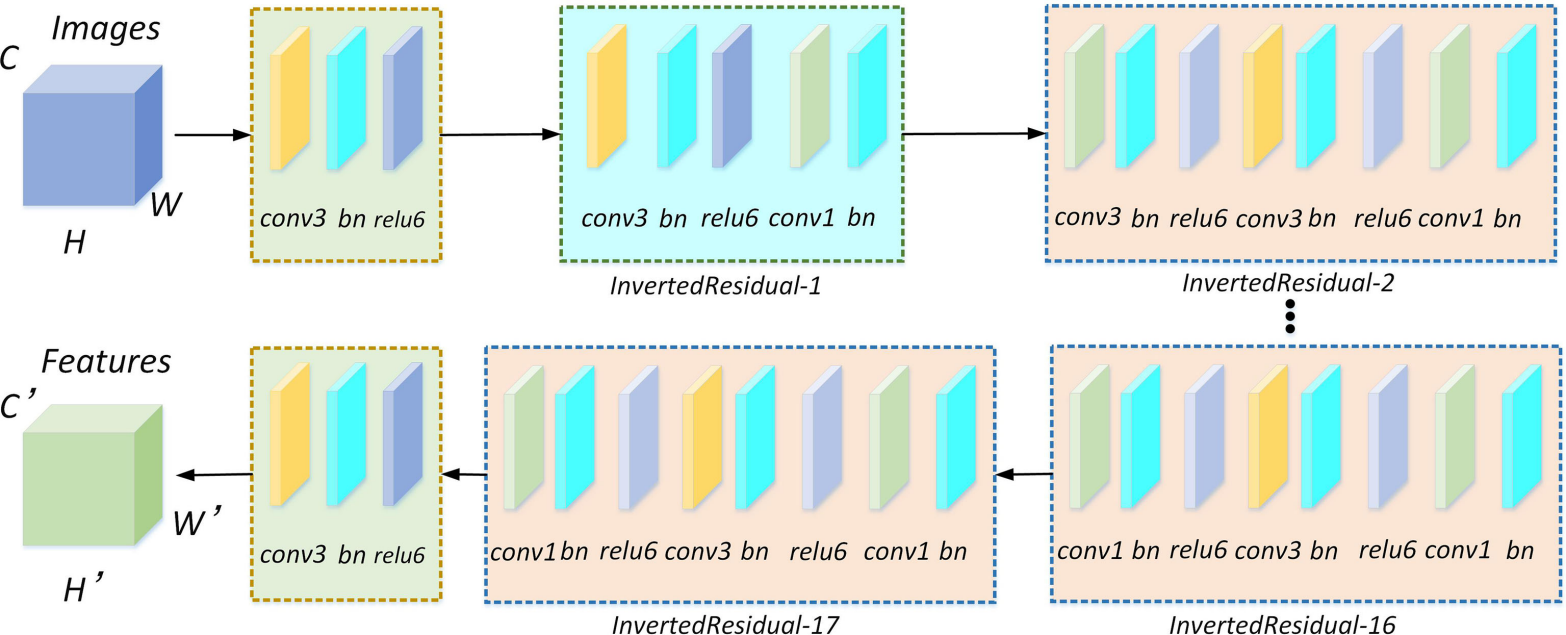
MobileNetV2 network for feature extraction. There are 17 inverted residual modules included in the network. Each inverted residual module consists of a depthwise separable convolution, and the depthwise separable convolution is composed of 1×1 and 3×3 standard convolutions, batch normalization, and relu6 activation functions.

In the study, the higher segmentation speed lightweight MobileNetV2 network is selected to replace Xception according to the accuracy requirements of the leaf veins’ segmentation. The MobileNetV2 structure is shown in **Figure 4**. The network mainly comprises standard convolution, depthwise separable convolution, and the ReLU6 activation function. Among them, the inverted residual structure is the most critical module in the network.

1. Through a feature extraction network, the feature map can be obtained from the input image by standard convolution. Firstly, the 1×1 and 3×3 standard convolutions carry out feature map information perception through specified step size and receptive field. Then, the inner product of the feature map and the convolution kernel are performed to obtain the activation value of the target. Finally, feature extraction can be achieved through channel stacking. Feature extraction is calculated using the following formula (1),


(1) $${\text{Y}}_{\left(\text{i},\text{j}\right)}={\left[X⊗Z\right]}_{\left(\text{i},\text{j}\right)}=\sum_{c=1}^{\text{C}}\sum_{\text{m}=\text{l}}^{\text{K}}\sum_{\text{n}=1}^{\text{K}}\left[X_{c}(S_{i}+\text{m},S_{j}+\text{n})W_{c}(\text{m},\text{n})\right]$$

where, *X*, the input feature information, and *Y*, the output feature information of the image after the convolution operation; (*i*,*j*) , the size of the feature map; ⊗ , the convolution operation; *c*, *m*, and *n*, intermediate variables; *C*, the feature channel; *K*, the size of the convolution kernel; *S*, the convolution stride; *Z*, the convolution kernel.

2. Many depthwise separable convolutions are used in MobileNetV2 to reduce model parameters and the occupancy rate of computer memory resources.

**Figure 5:**
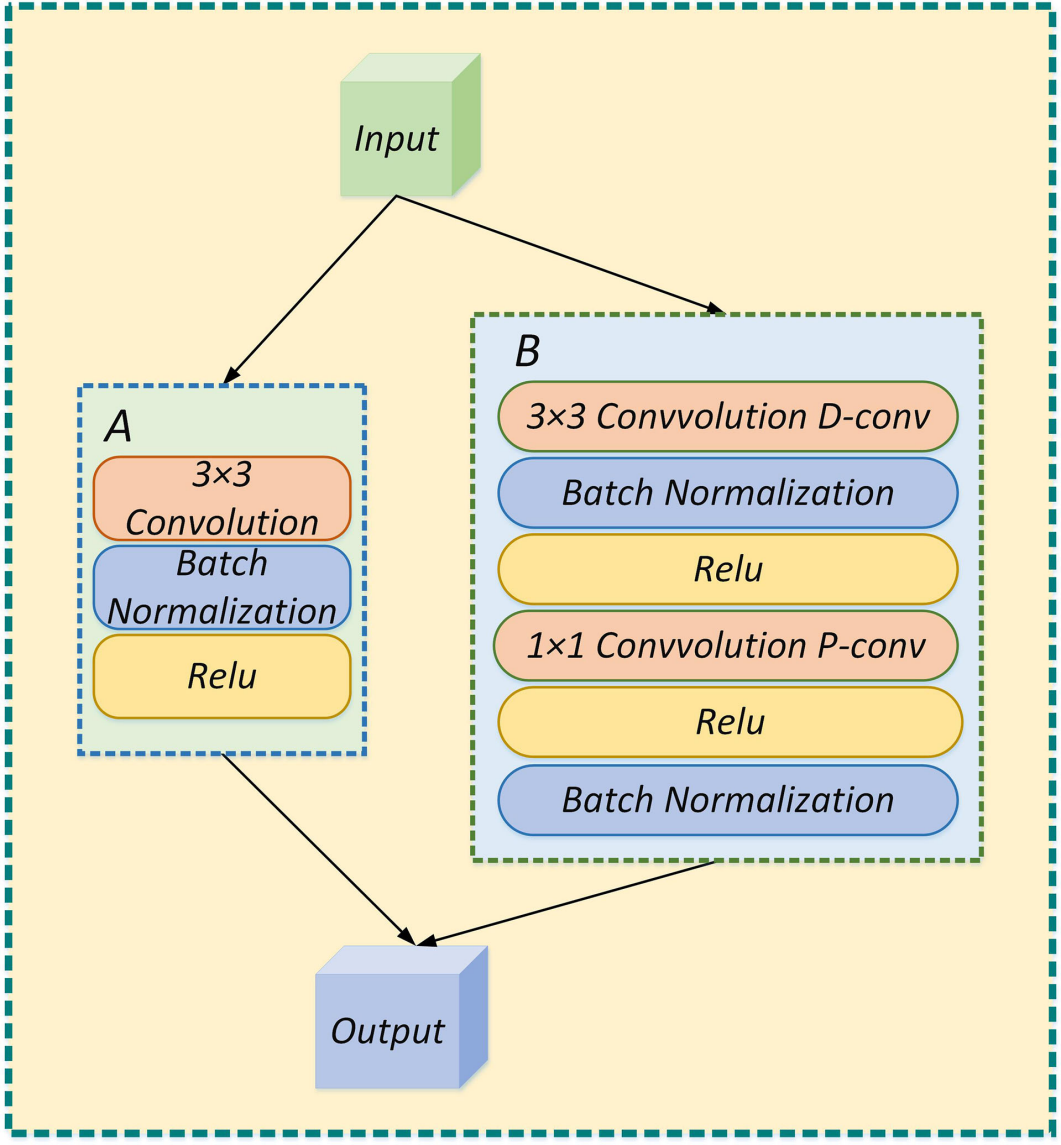
**(A)** The feature extraction process is performed by standard convolution; **(B)** The feature extraction process is performed by depthwise separable convolution.

As shown in **Figure 5**, the depthwise separable convolution is divided into a deep convolution and a pointwise convolution. Depthwise convolution processes each channel and the convolution kernel separately, among which the number of channels must equal the number of convolution kernels. Subsequently, the number of feature channels is recovered by a pointwise convolution operation with 1×1 convolution.

**Figure 6:**
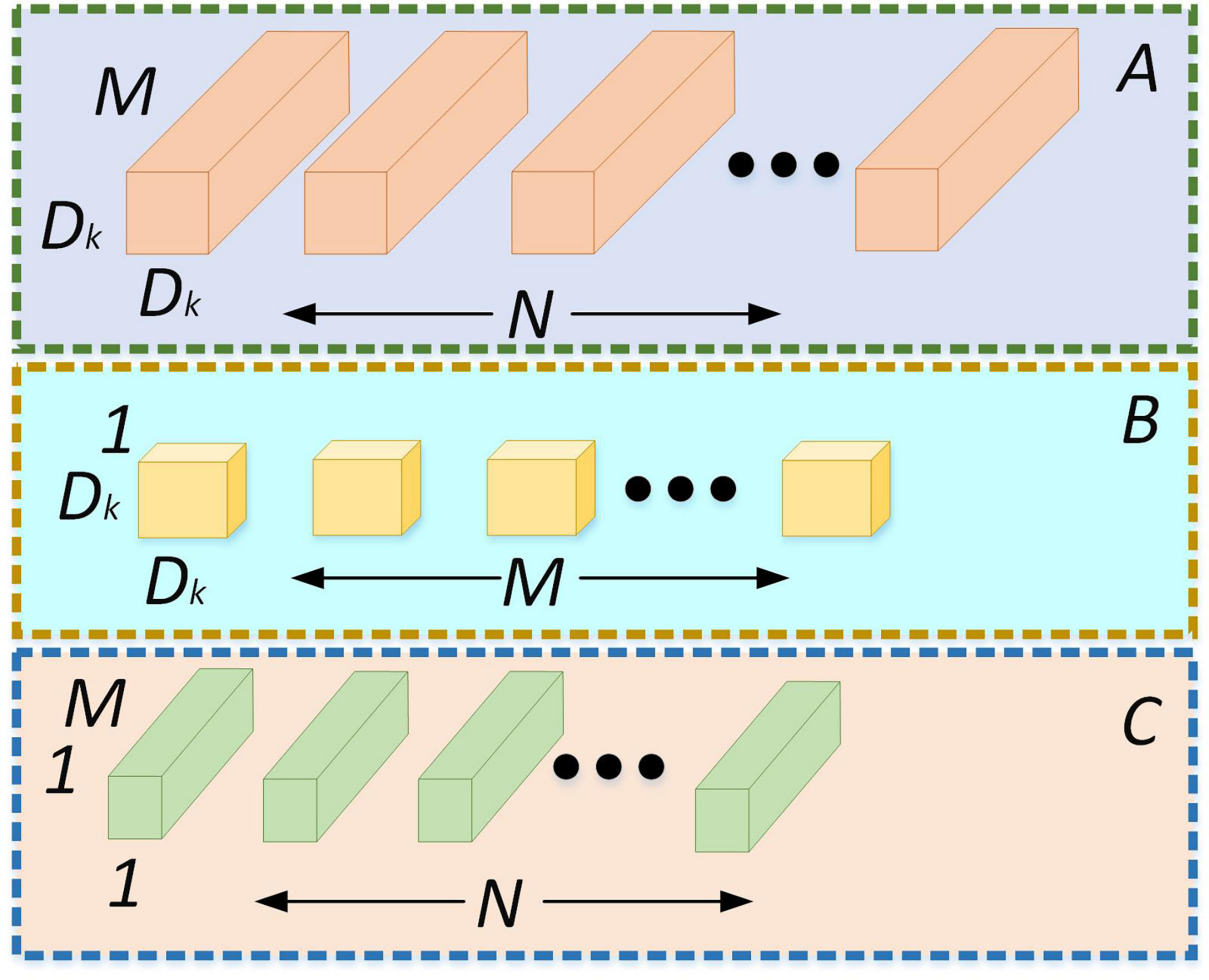
**(A)** standard convolution; **(B)** deep convolution; **(C)** pointwise convolution.

Standard convolution and depthwise separable convolution model parameters are analyzed. As shown in **Figure 6**, *D*_*F*_×*D*_*F*_×*M* , the size of the input feature map; *D*_*F*_×*D*_*F*_×*N* , the size of the output feature map. Among, *D*_*F*_ , the height and width of the feature map; *M* and *N*, the numbers of channels; *D*_*K*_×*D*_*K*_ , the size of the convolution kernel.

The calculation amount of standard convolution is calculated using the following formula (2),


(2) $$Data_{conv}=D_{K}\times D_{K}\times \text{M}\times D_{F}\times D_{F}\times \text{N}$$

The calculation amount of depthwise separable convolution is calculated using the following formula (3),


(3) $$Data_{DSC}=D_{K}\times D_{K}\times \text{M}\times D_{F}+\text{M}\times D_{F}\times D_{F}\times \text{N}$$

The ratio of the calculation amount of standard convolution and depthwise separable convolution is calculated using the following formula (4),


(4) $$\text{ζ}=\frac{Data_{conv}}{Data_{DSC}}=\frac{D_{K}\times D_{K}\times \text{M}\times D_{F}\times D_{F}\times \text{N}}{D_{K}\times D_{K}\times \text{M}\times D_{F}+\text{M}\times D_{F}\times D_{F}\times \text{N}}=\frac{1}{N}+\frac{1}{D_{K}^{2}}$$

If a 3×3 convolution kernel is used, the calculation amount of depthwise separable convolution is only about one-ninth of standard convolution.

3. A ReLU activation function is usually connected to alleviate the overfitting problem in convolutional neural networks. To implant a MobileNetV2 network into mobile devices, the activation value is limited to (0, 6). On the mobile device, float18/INT8 can satisfy the resolution and reduce the occurrence of gradient disappearance. The ReLU6 calculation formula is calculated using the following formula (5),


(5) F(x)=Min(Max(0,x),6)

In the above formula, *x*, the input feature map; *Max* , the upper limit of the restricted range; *Min* , the lower limit of the restricted range; *F*(*x*) , the output feature map; (0, 6), the output range of the ReLU6 activation function. So the derivative is also 0 when *x* is more than 6.

**Figure 7:**
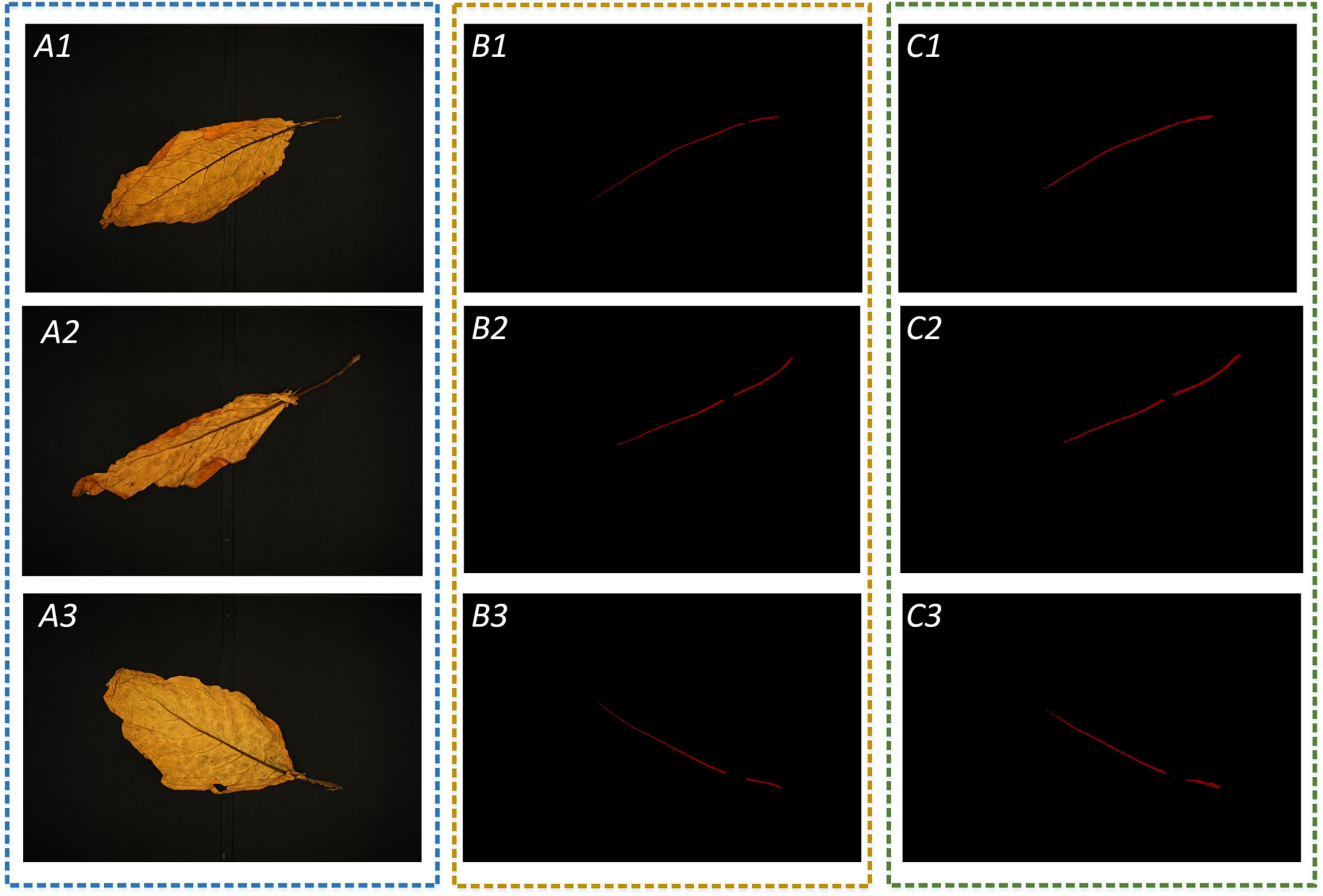
Test the result of leaf vein segmentation; A1, A2, and A3 are three images of flue-cured tobacco chosen at random; B1, B2, and B3 are manual annotations of leaf vein regions; and C1, C2, and C3 are the results of leaf vein segmentation using the MobileNetV2-DeepLabV3+.

The MobileNetV2-DeepLabV3+ is proposed in this study to perform leaf veins’ segmentation. MobileNetV2 is used for the feature extraction network, and the ASPP structure is used to obtain more in-depth semantic feature information. Finally, the shallow and deep feature information is fused to obtain the final segmentation result. Based on the semantic segmentation network, a large number of datasets can be calibrated, and then the supervised learning method is used to extract the target feature information. In this study, 512×512 pixels RGB images are entered into the model for training by the transfer learning method. The segmentation results are shown in **Figure 7**. Leaf veins are segmented accurately in the mobileNetV2-DeepLabV3+ network. However, some leaf veins are segmented incompletely due to leaf folds occasionally, which needs to be further optimized.

#### 2.2.3 Convex Hull-Scan method

Due to wrinkles on the leaves, the leaf vein segmentation is incomplete, which affects the accuracy of related parameter measurements. This study introduces the Convex Hull-Scan method to repair and obtain more complete leaf veins. The implementation process of the Convex Hull-Scan method is as follows.

**Figure 8:**
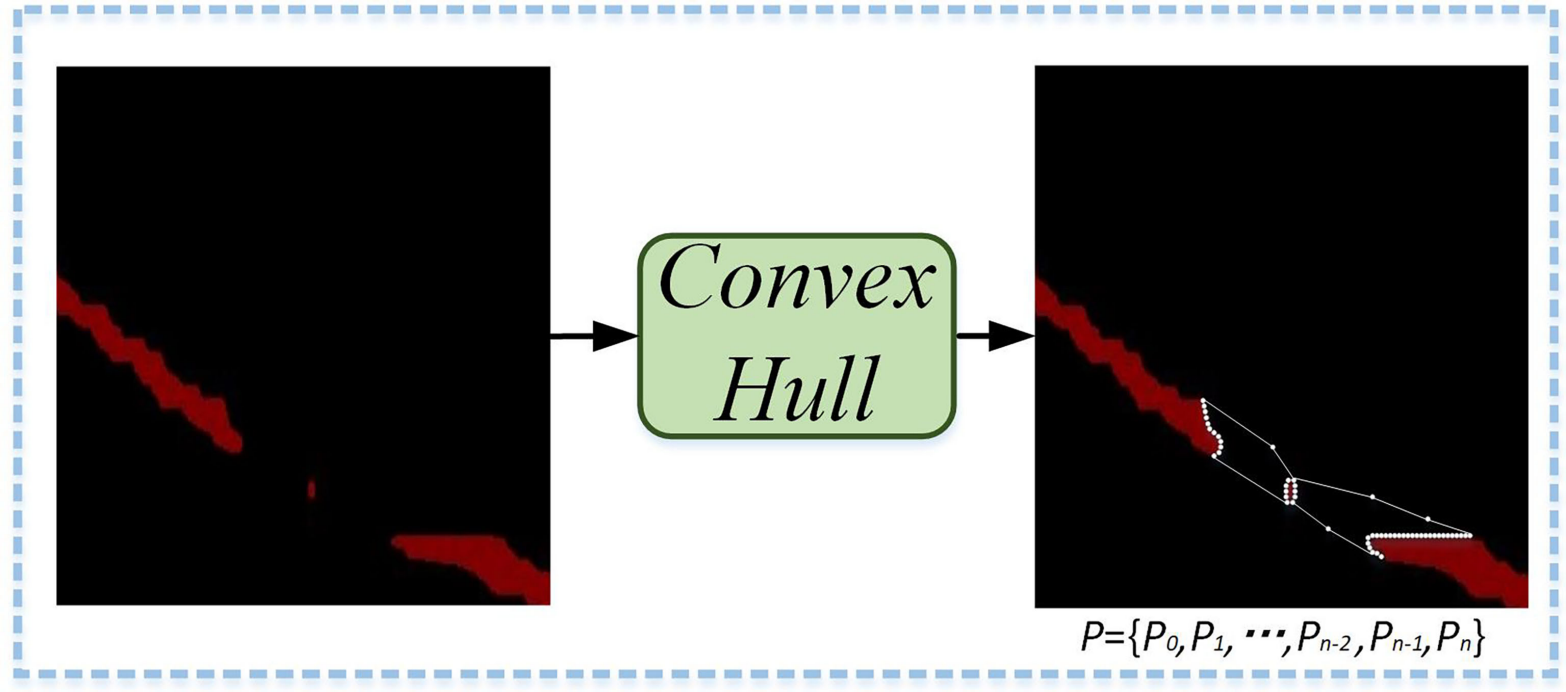
Minimum convex polygonal construction diagram. A minimum convex polygon is formed by the edge vertices of the leaf veins’ incomplete segmentation regions, the noise points between regions, and small isolated regions.

Firstly, Graham’s method in Convex Hull is used to find the incomplete segmentation regions of leaf veins. Convex polygon point sets (P) are formed by the edge vertices of the leaf veins’ incomplete segmentation regions, the noise points between regions, and small isolated regions. As shown in **Figure 8**, the Convex Hull method constructs the minimum convex polygon.

The specific implementation process of Graham’s method to find the minimum convex polygon vertices was as follows.

**Figure 9:**
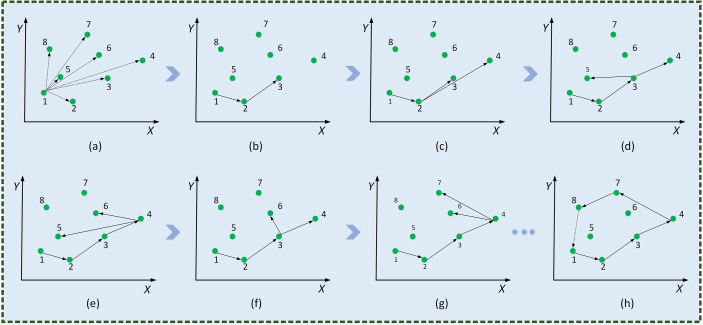
Minimum convex polygonal construction diagram. Traverse coordinate points **(A)**, $\vec{P_{1}P_{2}}\times \vec{P_{1}P_{3}}>0$ , deposit *P*_3_ **(B)**, $\vec{P_{2}P_{3}}\times \vec{P_{2}P_{4}}>0$ , deposit *P*_4_ **(C)**, $\vec{P_{3}P_{4}}\times \vec{P_{3}P_{5}}>0$ , deposit *P*_5_ **(D)**, $\vec{P_{4}P_{5}}\times \vec{P_{4}P_{6}}<0$ , pop up *P*_5_ **(E)**, $\vec{P_{3}P_{4}}\times \vec{P_{3}P_{6}}>0$ , deposit *P*_6_ **(F)**, $\vec{P_{4}P_{6}}\times \vec{P_{4}P_{7}}<0$ , pop up  *P*_6_ **(G)**,…, form the smallest convex polygon **(H)**.

(1) The minimum value on all x-axes is found through the image coordinate system in the leaf veins’ occlusion regions. If there are multiple identical values on the x-axis, it takes the minimum value on the y-axis, so that the starting coordinate point, *P*_1_(*x*_*min*_,*y*_*min*_) , is determined.
(2) All coordinate points are sorted counterclockwise according to polar angles. Precedence is given to the nearest coordinates to the starting point when the polar angles are equal.
(3) The minimum convex hull formation process is shown in **Figure 9**. An array, *ARR*[*N*] , is used to store the set of Convex Hull points in leaf veins’ occlusion regions. Firstly, *P*_1_ and *P*_2_ are stored in an array. Subsequently, all coordinate points of the Convex Hull point set are scanned. The point *P*_*i*_ is determined on the periphery of the array by the cross-product between *ARR*[*N*] and *ARR*[*N*−1] .

The implementation formula is calculated using the following formula (6),


(6) $$Dat=ARR[\text{N}]\times P_{i}-ARR[\text{N}]\times ARR[\text{N}-1]-ARR[\text{N}-1]\times P_{i}+{\left\{ARR[\text{N}-1]\right\}}^{2}$$

where, if *Dat*>0 , it is the set of points inside the convex packet, and if *Dat*<0 , it is the set of vertices of the convex packet.

**Figure 10:**
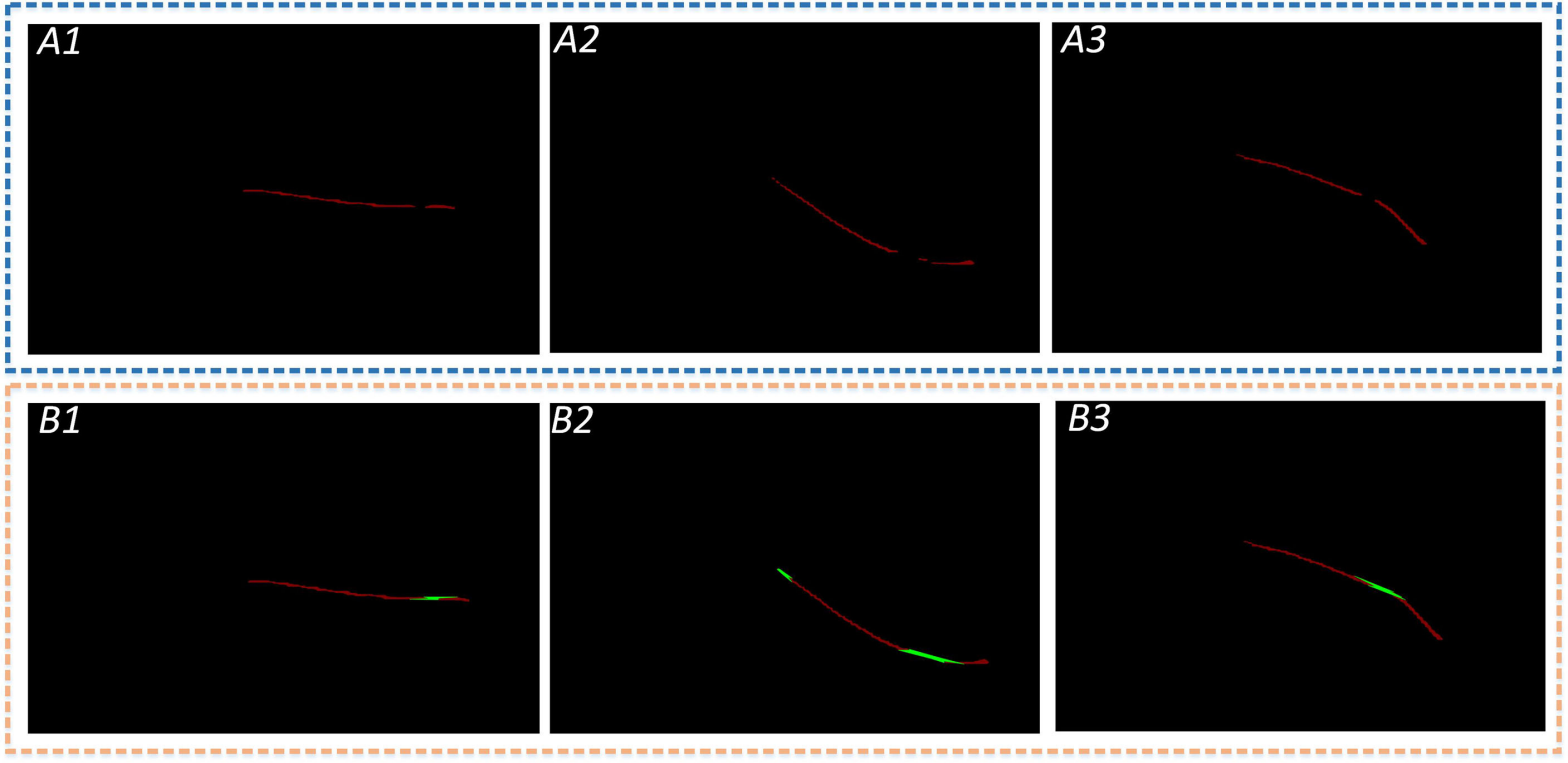
Leaf veins repair images. The A1, A2, and A3 show that the segmented leaf vein regions pass through MobileNetV2-DeepLabV3+, and all three images have leaf vein occlusion, resulting in incomplete segmentation; B1, B2, and B3 show that the leaf veins were repaired using the Convex Hull-Scan method.

Subsequently, the set *P* of all points is ordered into the least convex polygon. According to the permutation formula, all coordinate points are scan-connected using the Euclidean distance to fill incomplete segmentation regions. As shown in **Figure 10**, a complete leaf vein is obtained by using the Convex Hull-Scan method.

### 2.3 Leaf veins parameters measurement

This section illustrates the leaf veins parameter measurement in detail. The length measurement is introduced in Section 2.3.1, and the width measurement is elaborated in Section 2.3.2.

Length and width parametric indicators are extracted based on the repaired leaf veins. This paper does more fine-grained processing for leaf veins to increase the accuracy of leaf vein measurement. An F-3MS refinement algorithm is proposed to extract leaf veins’ skeleton lines.

#### 2.3.1 Length measurement

The repaired leaf veins needed to be preprocessed through grayscale, binarization, and nonlinear filtering to measure the leaf veins’ width. And then, a single-pixel skeleton line is obtained by the Morphological Skeleton refinement algorithm. The length of the leaf veins is measured indirectly through the number of pixel points. However, there are still fine cavities and slightly rough contours in the repaired regions of leaf veins. Moreover, there were many burrs in leaf veins’ skeleton lines by ordinary refinement algorithms, resulting in low parameter measurement accuracy. In this research, an F-3MS refinement algorithm is proposed, which can reduce the burrs’ appearance of skeleton lines. The specific implementation process of the algorithm is as follows.

(1) Firstly, the Flood Fill Algorithm is put forward to fill the background of the segmented image with white. Subsequently, the NOT operation is used to fill the image with the Flood Fill Algorithm. Finally, the OR operation is performed on the original image with the NOT operation image. Therefore, these fine holes inside the leaf veins are filled in three steps.

(2) These isolated dots in leaf vein regions are eliminated by open operation.

(3) Leaf vein images are processed with three different weights for RGB to obtain a gray image. The gray processing formula is calculated using the following formula (7),


(7) Gray=0.299×R+0.587×G+0.114×B

where R, G, and B represent the values of the three primary colors—red, green, and blue—of the leaf veins segmentation image. “Gray” is a grayscale value.

(4) To preserve more leaf vein details, median filtering is used to smooth leaf veins’ contours and remove noise points.

(5) The maximum interclass variance method is used to binarize images. This principle is shown below.

The pixel value of the leaf vein image is divided into [1,2,⋯,*l*] levels, and *n*_*i*_ is used to represent the number of certain image values. So the total pixels’ value of the leaf veins image is (8),


(8) $$\text{N}=n_{1}+n_{2}+\cdots +n_{i}+\cdots +n_{l}$$

Where the frequency of a single pixel in the image is (9),


(9) $$p_{i}=\frac{n_{i}}{N_{i}},\;p_{i.}>0,p_{1}+p_{2}+\cdots +p_{n}=1$$

Define two variables as the sum of local frequency values, and the relationship is (10),


(10) $$w_{0}=\sum_{i=1}^{\text{k}}p_{i},w_{1}=\sum_{\text{i}=\text{k}+1}^{\text{l}}p_{i}$$

The image’s foreground and background frequencies are (11),


(11) $$u_{T}=\sum_{\text{i}=1}^{\text{l}}\text{i}*p_{i},u_{0}=\sum_{i=1}^{k}i*\frac{p_{i}}{\sum_{\text{i}=1}^{\text{k}}p_{i}},u_{1}=\sum_{\text{i}=\text{k}+1}^{\text{l}}\text{i}*\frac{p_{i}}{\sum_{\text{i}=\text{k}+1}^{\text{k}}p_{i}}$$

The relationship in the above equation can be described as (12),


(12) $$w_{0}u_{0}+w_{1}u_{1}=u_{T},w_{0}+w_{1}=1$$

(6) To measure leaf vein length, a single-pixel skeleton line is obtained by the Morphological Skeleton refinement algorithm. Hence, the F-3MS refinement algorithm is constructed.

#### 2.3.2 Width measurement

**Figure 11:**
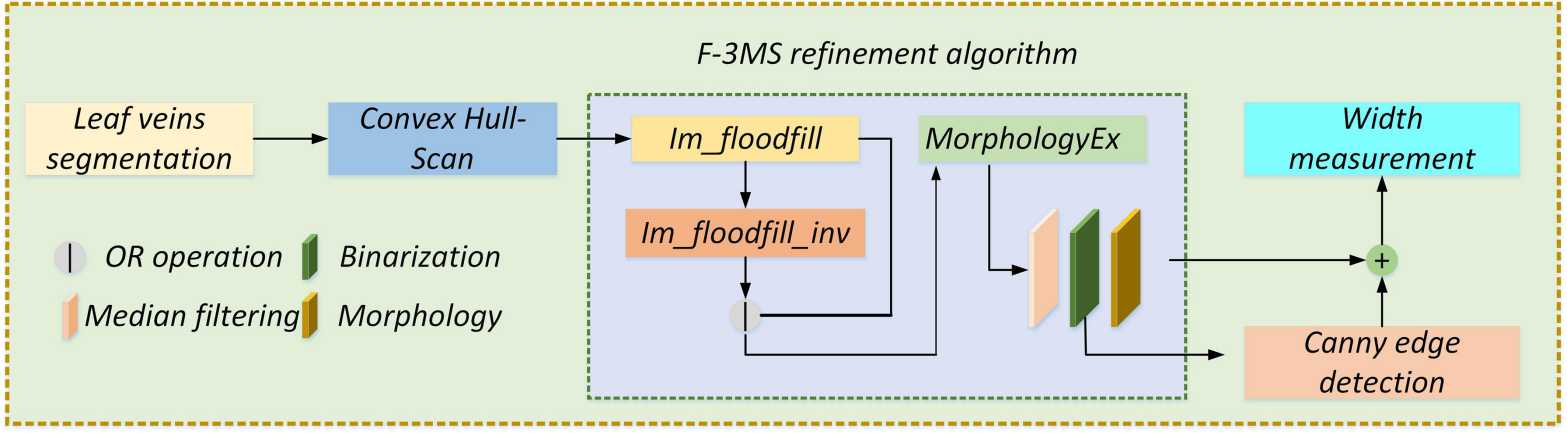
Width geometry model construction. Before measuring the leaf veins’ width, the skeleton line of the complete leaf veins needs to be extracted by the F-3MS refinement algorithm. At the same time, the edge detection algorithm extracts the leaf veins’ contour. Finally, by constructing a width geometric model and using the image bit operation, the width of the leaf veins is measured.

As shown in **Figure 11**, leaf veins’ contour can be obtained through the Canny edge detection after binarization. Subsequently, the AND operation is performed on the contour and skeleton line images to obtain the fusion image. Finally, the geometric model is constructed in the fused image to calculate the leaf veins’ width.

**Figure 12:**
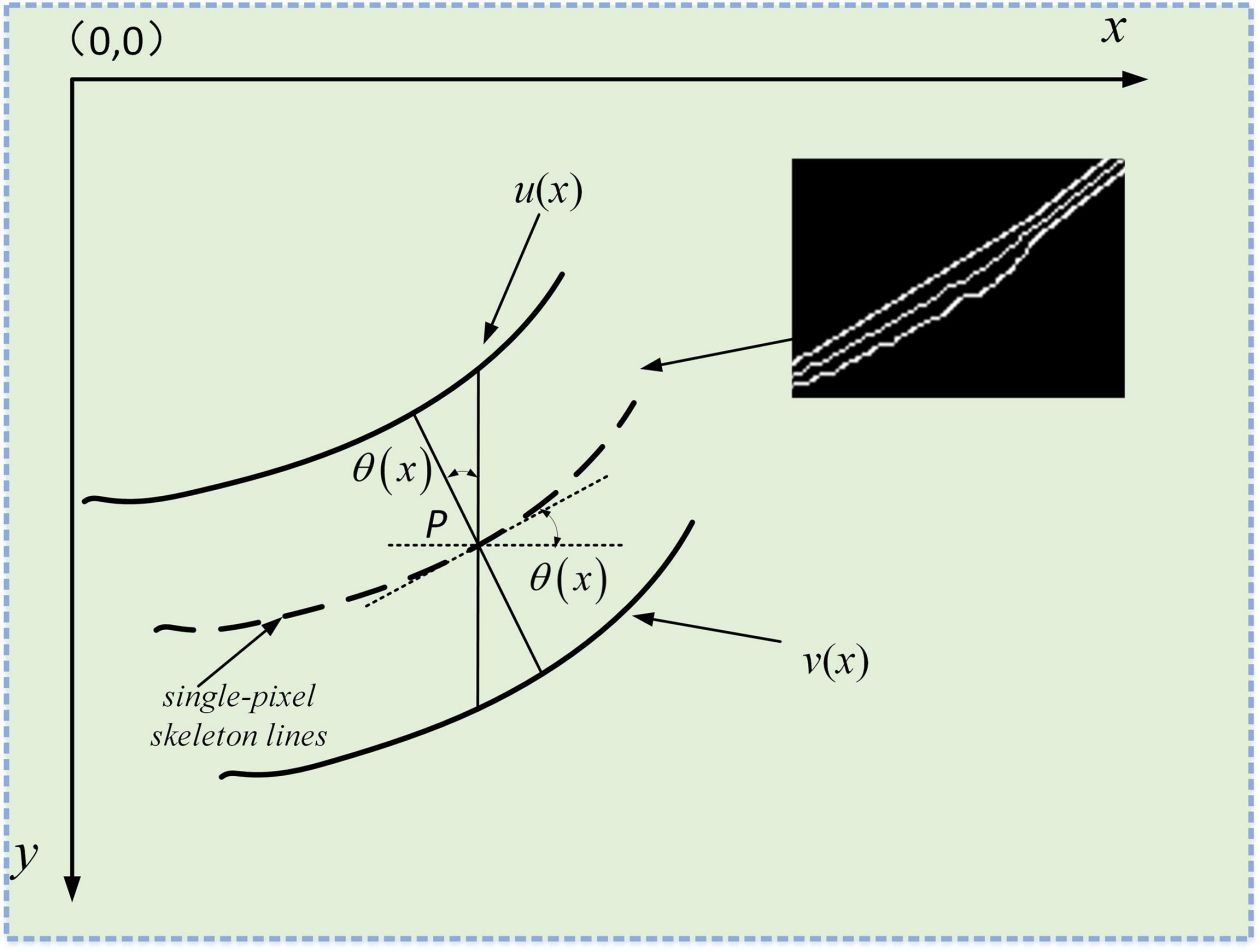
Measure the true width of the leaf veins. the screen coordinate system is established in the image. The solid lines *u*(*x*) and *u*(*x*) represent the upper and lower contours of the leaf veins, respectively, and the dashed lines represent the refined vein skeleton lines.

As shown in **Figure 12**, the leaf veins’ vertical distance is [*v*(*x*)−*u*(*x*)]/2 . However, in most cases, the vertical distance between the leaf veins cannot represent its true width, and the true width is less or equal to [*v*(*x*)−*u*(*x*)]/2 . The image coordinate system is used to denote any point P on the skeleton lines to obtain the true width of leaf veins, *P*_*i*_(*x*_*i*_,[*v*(*x*)−*u*(*x*)]/2) . Therefore, the front and back pixels coordinates of *P* are *P*_*i*−1_(*x*_*i*−1_,[*v*(*x*_*i*−1_)−*u*(*x*_*i*−1_)]/2) and *P*_*i*+1_(*x*_*i*+1_,[*v*(*x*_*i*+1_)−*u*(*x*_*i*+1_)]/2) , respectively.

The cosine of the angle *θ* can be calculated between the coordinates of two-pixel points according to the triangular geometry formula. The cosine of the angle *θ* formula is calculated using the following formula (13),


(13) $$\cos\theta =\frac{x_{i+1}-x_{i-1}}{\sqrt{{\left(x_{i+1}-x_{i-1}\right)}^{2}+{\left(\left[\text{v}({\text{x}}_{\text{i}+1})-\text{u}({\text{x}}_{\text{i}+1})\right]/2-\left[v(x_{i-1})-\text{u}(x_{i-1})\right]/2\right)}^{2}}}$$

Then the leaf vein’s true width passing through any point *P* on the skeleton lines can be expressed as (14),(15),(16),


(14) $$\left[\left(x_{1},\text{u}(x_{1})\right),\left(x_{2},\text{u}(x_{2})\right),\cdots ,\left(x_{i},\text{u}(x_{i})\right),\cdots ,\left(x_{m},\text{u}(x_{m})\right)\right]$$

(15) $$\left[\left(x_{1},\text{v}(x_{1})\right),\left(x_{2},v(x_{2})\right),\cdots ,\left(x_{i},\text{v}(x_{i})\right),\cdots ,\left(x_{m},\text{v}(x_{m})\right)\right]$$

(16) $$\left[\left(x_{1},\frac{v(x_{1})-u(x_{1})}{2}\right),\left(x_{2},\frac{v(x_{2})-u(x_{2})}{2}\right),\cdots ,\left(x_{i},\frac{v(x_{i})-u(x_{i})}{2}\right),\cdots ,\left(x_{m},\frac{v(x_{m})-u(x_{m})}{2}\right)\right]$$

And the vertical distance of each pixel coordinate passing through the leaf vein’s skeleton lines is (17),


(17) $$\left[\frac{v(x_{1})-u(x_{1})}{2},\frac{v(x_{2})-u(x_{2})}{2},\cdots ,\frac{v(x_{i})-u(x_{i})}{2},\cdots ,\frac{v(x_{m})-u(x_{m})}{2}\right]$$

The cosine angle is (18),


(18) $$\left[\cos\theta_{1},\cos\theta_{2},\cdots ,\cos\theta_{i},\cdots ,\cos\theta_{m}\right]$$

Therefore, the leaf vein’s true width through the coordinates of each point of the skeleton lines is (19),


(19) $$\left[\frac{v\left(x_{1}\right)-u\left(x_{1}\right)}{2}\cos\theta_{1},\frac{v\left(x_{2}\right)-u\left(x_{2}\right)}{2}\cos\theta_{2},\cdots ,\frac{v\left(x_{i}\right)-u\left(x_{i}\right)}{2}\cos\theta_{i},\cdots ,\frac{v\left(x_{m}\right)-u\left(x_{m}\right)}{2}\cos\theta_{m}\right]$$

## 3 Implementation details

This section illustrates implementation in detail. The experimental platform construction is introduced in Section 3.1. The network evaluation is elaborated in Section 3.2, and the train details used for the network’s performance are explained in Section 2.3.

### 3.1 Experimental platform construction

The experimental hardware for this study includes an Intel i5-12600KF CPU, and an NVIDIA GeForce RTX3060 GPU with 12 GB memory. A series of experimental operations are performed on Ubuntu20.04 L.S.T. Based on the PyCharm2020 open-source software, Python3.8.12 and Pytorch1.10.0 are used to build the model of plant leaf veins segmentation, and OpenCV4.1.12 is selected as the development platform to achieve leaf vein repair and parameter measurement.

### 3.2 Network evaluation

In this study, to evaluate the segmentation performance of the network, five standard metrics in semantic segmentation are selected: MIoU, mPA, FPS, FLOPs, and model parameters. MIoU and mPA are two standard metrics for semantic segmentation. It calculates the intersection and merging ratio of the two sets, which are the true (ground truth) and predicted (predicted segmentation) values in the semantic partitioning problem. The MIoU and mPA formulas are calculated as follows (20), (21),


(20) $$MIoU=\frac{1}{k+1}\sum_{\text{i}=0}^{\text{k}}\frac{p_{ii}}{\sum_{\text{j}=0}^{\text{k}}p_{ij}+\sum_{\text{jk}}p_{ji}-p_{ii}}$$

(21) $$mPA=\frac{1}{k+1}\sum_{\text{i}=0}^{\text{k}}\frac{p_{ii}}{\sum_{j=0}^{k}p_{ij}}$$

where, *k*+1, the total number of categories; *i*, ground truth; *j*, predicted segmentation.

The MIoU and mPA formulas are equivalent to formulas (22), (23),


(22) $$MIoU=\frac{1}{\text{k}+1}\sum_{\text{i}=0}^{\text{k}}\frac{TP}{FN+FP+TP}$$

(23) $$mPA=\frac{1}{\text{k}+1}\sum_{\text{i}=0}^{\text{k}}\frac{TP+TN}{FN+FP+TP+TN}$$

where, TP, true positive; TN, true negative; FP, false positive; FN, false negative.

**Figure 13:**
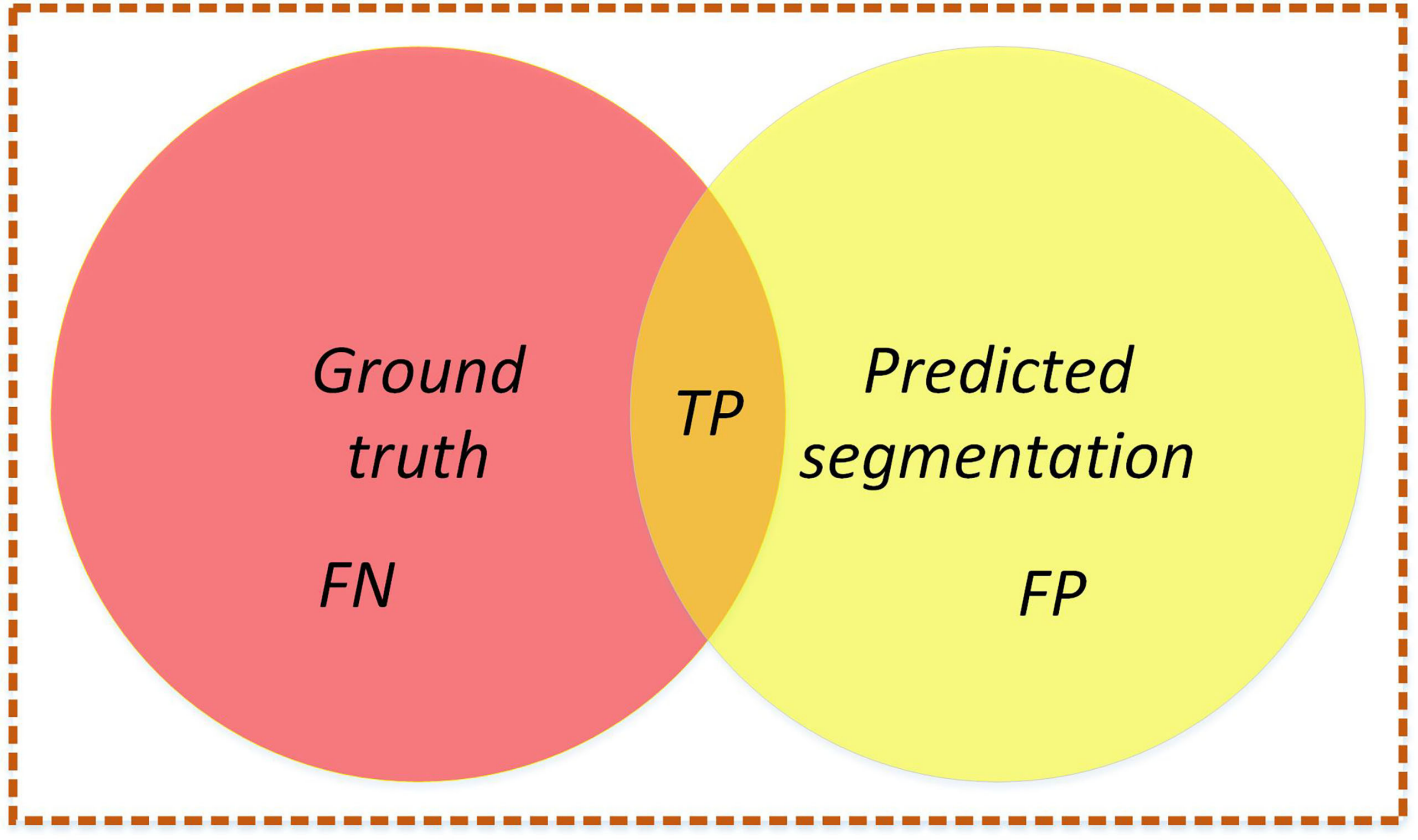
Intersection graph. The red circle represents the ground truth, the yellow circle represents the predicted segmentation, and the orange part is the intersection of the two circles. Calculate the ratio between the intersection of two circles (the orange part) and the union of two circles (red+orange+yellow). Ideally, the two circles coincide, and the ratio is 1.

Intuitive understanding is shown in **Figure 13**.

In this study, the leaf vein is a positive class(1), and the background is a negative class(0). A detailed description is shown in **Table 1**.

**Table 1:** Leaf veins segmentation confusion matrix.

Ground truth	Predicted segmentation	
	Leaf veins (1)	Background (0)
Leaf veins (1)	TP (true positive)	FN (false positive)
Background (0)	FP (true negative)	TN (false negative)

The model segmentation speed is evaluated in frames per second (FPS). Model parameters evaluate the training speed and memory resource usage of the model. FLOPs are floating point operations per second. It is worth noting that FPS is the number of images segmented per second during the testing phase.

### 3.3 Train details

This study uses transfer learning to train the network in the leaf vein segmentation stage. The network is trained for 100 epochs. To allow the network to train the dataset properly, the size of the image in the input network is 512×512 pixels. To prevent the influence of image data on the network, the training and validation sets are randomly shuffled before being input to the model. The network’s gradient decreases through iterative algorithms to decrease the learning rate, and better segmentation performance can be obtained ultimately.

The model’s parameters are updated using the Adaptive Moment Estimation (Adam) optimizer: the initial learning rate is 0.001, the “cos” learning scheduler is selected to adjust the learning rate, the weight decay is 5e−4, β1 = 0.9, β2 = 0.99, and the gamma is 0.94. Due to computer hardware and graphics card memory limitations, the feature extraction network is frozen for training at the first 50 epochs with a mini-batch size of 8, and the entire network is loaded for training after 50 epochs with a mini-batch size of 4. Additionally, the dropout size is set to 0.5. The process of updating model parameters during network training is shown in **Table 2**.

**Table 2:** Pseudo-code for the process of updating model parameters during network training.

*1. Initialization: learning rate (lr)*
*2. Initialization: Smoothing constant (decay rate)* *β*_1_ *and* *β*_2_ *for smoothing m and v, respectively.*
*3. Initialization: Parameters that can be learned θ*_0_
*4. Initialization: m*_0_ *=0*, *v*_0_ *=0, t=0*
*5. while doing:* *(No stopping training)*
*6. Number of training sessions updated: t+=1*
*7. Calculated gradients: g*_*t*_ *(All learnable parameters have their own gradients, so g*_*t*_ *represents the set of all gradients)*
*8. Cumulative gradient: m*_*t*_=*β*_1_*m_*t*−1_+(1−*β*_1_)*g_*t*_ *(Each derivative corresponds to an m, so m is also a set.)*
*9. Square of cumulative gradient: v*_*t*_=*β*_2_*v_*t*−1_+(1−*β*_2_)*(*g*_*t*_)^2^ *(Each derivative corresponds to a v, so v is also a set)*
*10. Deviation correction m:* ${\hat{m}}_{t}=\frac{m_{t}}{1-{\left(\beta_{1}\right)}^{t}}$
*11. Number of training sessions updated:* ${\hat{v}}_{t}=\frac{v_{t}}{1-{\left(\beta_{2}\right)}^{t}}$
*12. Number of training sessions updated:* $\theta_{t}=\theta_{t-1}-\frac{{\hat{m}}_{t}}{\sqrt{{\hat{v}}_{t}+\epsilon }}*lr$
*13. end while*

## 4 Results

This section illustrates the results in detail. The network fine-tuning is in Section 4.1. Comparison with other networks is elaborated in Section 4.2, and the parameters’ measurement verification is analyzed in Section 4.3.

### 4.1 Network fine-tuning

In neural networks, when the parameters are selected differently, the training results will be significantly affected. For better performance of leaf vein segmentation, fine-tuning the network’s parameters is essential. Given initial learning, the rate is 0.001, and the individual parameters of the network are fine-tuned, as shown in **Table 3**. This study compares the tuning strategy of the learning rate, the optimizer’s choice, the multiplicity of feature map downsampling, and whether focal_loss is used. Firstly, the “step” and “cos” learning schedulers are selected, and using “cos” is higher than using “step” for MIoU and mPA at 2.7% and 2.59%, respectively. Secondly, the SGD and Adam optimizers are often selected to train deep convolutional neural networks. Compared to the SGD optimizer, using the Adam optimizer, MIoU and mPA reach 81.50% and 92.89%, respectively. Meanwhile, the feature map is downsampled 8 times when the network is trained, and the performance of the segmentation is better. Finally, Focal_loss is added to the network, but the segmentation performance is not very good. Therefore, when training the network, this study chooses the “cos” learning scheduler, Adam optimizer, and 8 times downsampling. The training effect of the network model is relatively excellent.

**Table 3:** Network segmentation performance comparison for different parameters selection.

Number	Learning scheduler		optimizer		Downsample_factor		Focal_loss	MIoU(%)	mPA(%)
	step	cos	SGD	Adam	8	16			
①	✓		✓		✓			74.15	87.66
②		✓	✓		✓			76.85	90.25
③		✓		✓	✓			81.50	92.89
④		✓		✓		✓		81.49	92.51
⑤		✓		✓	✓		✓	81.39	92.87

Based on **Table 3**, different learning rates are chosen and the results are shown in **Table 4**. Similarly, when the initial learning rate is set to 0.001, the training result of the network model is optimal.

**Table 4:** Comparison of network training at different learning rates.

Number	Learning rate	MIoU (%)	mPA (%)
①	0.05	81.36	92.57
②	0.01	81.45	92.19
③	0.005	81.39	92.60
④	0.001	81.50	92.89
⑤	0.0005	81.46	92.74
⑥	0.0001	81.48	92.89

**Figure 14:**
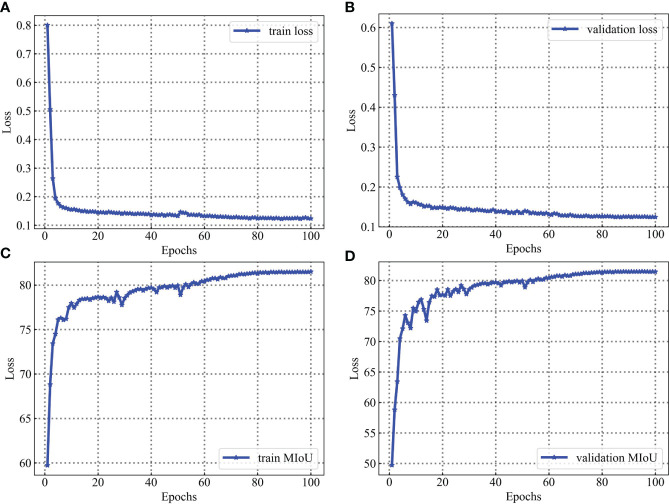
Training and validation process for the leaf vein dataset. Parameters are evaluated after each epoch. **(A)** is loss evaluated on the training sets. **(B)** is loss evaluated on the validation sets. **(C)** is MIoU evaluated on the training sets. **(D)** is MIoU evaluated on the validation sets.

For the leaf vein segmentation, although a smaller dataset is used, the results of this training are very satisfactory, as shown in **Figure 14**. During the training process, the network model converged rapidly in the first 60 epochs and gradually stabilized after 40 epochs. The high performance of segmentation can be attributed to two main reasons. Firstly, professional industrial cameras are used to capture the images at the time of data acquisition, which greatly reduces the influence of variations in environmental lighting on the captured images and makes the image data distribution relatively consistent, and it is possible to make the training model more robust with a smaller training data set. This has been investigated as a key for agricultural applications. Secondly, in addition to the use of specialist equipment to acquire image data, the use of data augmentation and network fine-tuning also has an extremely significant impact on the final trained model. Because data augmentation can prevent overfitting of the model, and network fine-tuning can greatly take advantage of the performance of the model.

### 4.2 Comparison with other networks

DeepLabV3+ is compared with the following baselines, which consist of three feature extraction networks: Xception, ResNet50, and MobileNetV2.

**Xception** (Chollet, 2017): Xception is an improved network of InceptionV3 proposed by Google after Inception. The main improvement is to use depthwise separable convolution to replace the multi-size convolution kernel feature response operation in Inception v3.

**ResNet5**0 (He et al., 2016): ResNet has been used in a wide variety of feature extraction applications. The number of layers of the deep learning network is greater and theoretically more expressive. However, the CNN network reaches a certain depth and then deepens, and the classification performance does not improve. At the same time, it causes the network to converge more slowly and the accuracy rate to decrease. Even if the dataset is increased to solve the over-fitting problem, the classification performance and accuracy will not improve. Hence, a residual structure in resnet50 was proposed to solve this problem of gradient disappearance and deep network training difficulty.

**MobileNetV2** (Sandler et al., 2018): The MobileNetV2 network was proposed by the Google team in 2018 and is more accurate and has a smaller model compared to the MobileNetV1 network. There are three main advantages in this network: inverted residuals, linear bottlenecks, and ReLU6 activation functions.

This study uses the above three networks as feature extraction networks. As shown in **Table 5**, MobileNetV2 as a feature extraction network has the highest MIoU and mPA, and the number of model parameters is only 5.813M, with a segmentation speed of 9.81 frames per second.

**Table 5:** Compare the segmentation performance of different feature extraction networks.

Network	MIoU (%)	mPA (%)	Params (M)	FPS	FLOPs (G)
Xception	79.51	90.1	54.709	5.55	83.420
ResNet50	73.34	83.11	39.634	6.52	48.673
MobileNetV2	81.50	92.89	5.813	9.81	39.962

In this study, to compare the segmentation performance of different networks, UNet (Ronneberger et al., 2015), PSPNet (Zhao et al., 2016), DeepLabV3 (Chen et al., 2017), DeepLabV3+ (Chen et al., 2018), LRASPP (Howard et al., 2019), HRNet (Sun et al., 2019), ResUNet (Fid et al., 2020), CGNet (Wu et al., 2021), TransUNet (Chen et al., 2021), and MobileNetV2-DeepLabV3+ (Ours) are all selected to train network. For different objects, the evaluation indicators of each network model are not consistent. MIoU, Params, FPS, and FLOPs are more widely recognized evaluation indicators for semantic segmentation. After training for 100 epochs, **Table 6** displays the experimental results. Although LRASPP and CGNet have smaller network model parameters and lower FLOPs, the network has faster training segmentation speed, the segmentation accuracy of the network is lower, which does not meet the actual production requirements. TransUNet has the highest MIoU, but the network has a large number of model parameters, the training speed is slow, and the segmentation speed is only 1.46 frames per second. In addition, during the training of TransUNet, the hardware configuration of the computer is also very high. However, the MobileNetV2-DeepLabV3+ performs faster segmentation speed and smaller model parameters, while it can meet actual production requirements.

**Table 6:** Comparison with other networks.

Network	mIOU (%)	Params (M)	FPS	FLOPs (G)
UNet	82.50	24.891	7.42	225.836
PSPNet	73.11	46.707	6.99	59.213
DeepLabV3	73.93	60.991	3.12	251.815
DeepLabV3+	79.51	54.709	5.55	83.420
LRASPP	64.70	3.218	21.58	2.073
HRNet	75.27	29.538	7.98	45.462
CGNet	64.00	0.493	34.67	1.468
ResUNet	79.68	43.933	8.72	92.050
TransUNet	88.68	93.192	1.46	128.677
Ours	81.50	5.813	9.81	39.962

### 4.3 Parameters measurement verification

**Figure 15:**
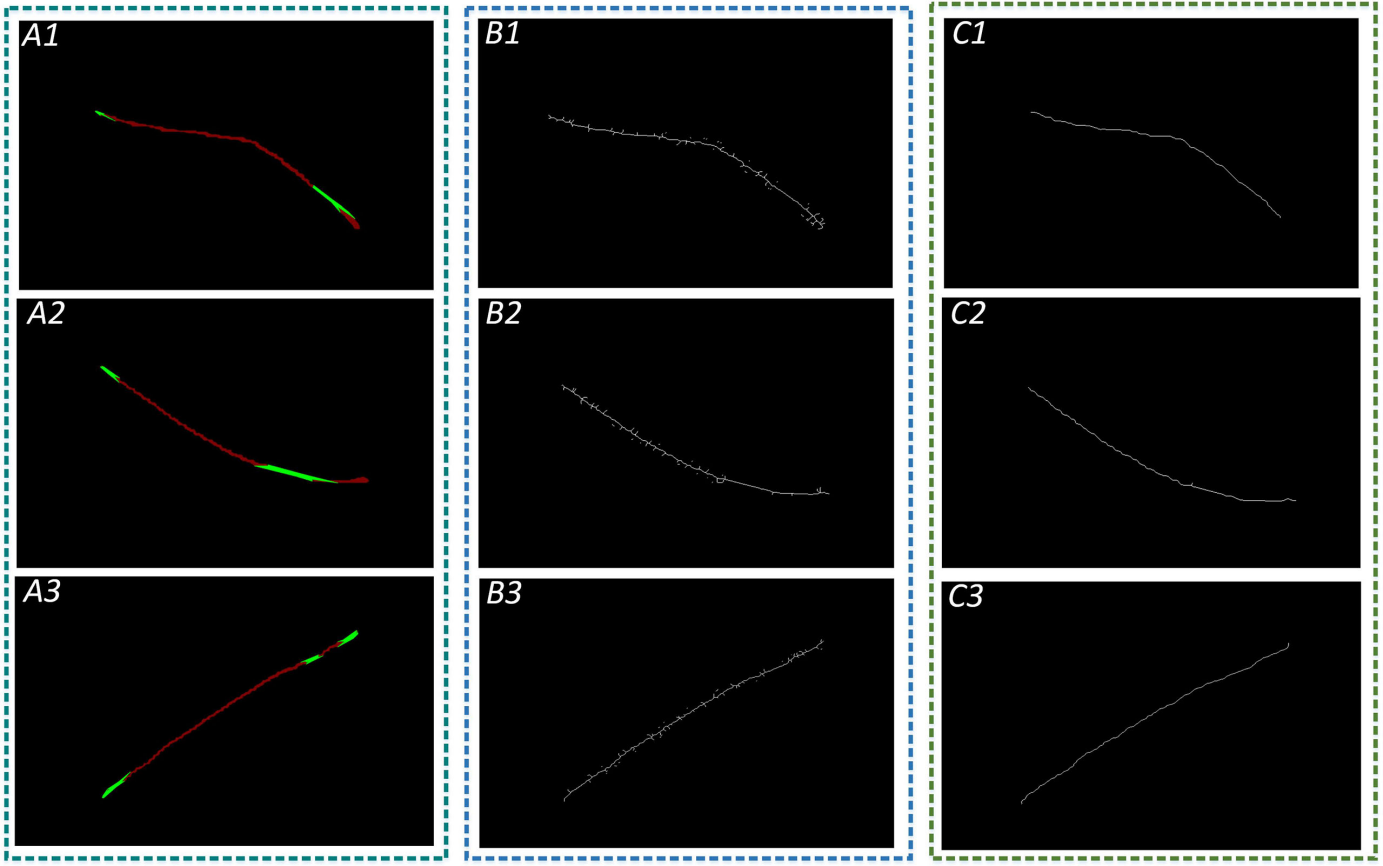
Refinement of leaf vein images. A1, A2, and A3 are images of restored leaf veins using the Convex Hull-Scan method; B1, B2, and B3 are leaf vein skeleton lines extracted using the Morphology Skeleton refinement algorithm; and C1, C2, and C3 are leaf vein skeleton lines extracted using the F-3MS algorithm.

The F-3MS and Morphological Skeleton refinement algorithms are used to extract skeleton lines from repaired leaf veins. The refinement effect of skeleton lines is shown in **Figure 15**.

Median filtering is processed with the convolution kernel sizes of 1×1, 3×3, 5×5, and 7×7 for the two refinement algorithms, respectively, and then the leaf vein length and width are measured. The measurement accuracy is shown in **Table 7**.

**Table 7:** Comparison of accuracy of leaf vein parameter measurement.

Algorithm	1 × 1		3 × 3		5 × 5		7 × 7	
	Length	Width	Length	Width	Length	Width	Length	Width
Morphological Skeleton	40.7955	21.1662	84.0921	69.7683	98.6972	78.7432	95.5889	80.3382
F-3MS	86.0089	76.8277	99.4463	85.4013	96.3642	96.1358	95.2083	87.9258

It can be seen from **Table 7** that the Morphological Skeleton refinement algorithm and the F-3MS refinement algorithm maintained high accuracy when using convolution kernels of 5×5 and 7×7 in the leaf vein length measurement. However, the F-3MS refinement algorithm used in this paper to process leaf veins indicates a higher accuracy rate. This study conducted a comprehensive performance comparison and selected the 5×5 convolution kernel to filter the leaf vein images. In measuring leaf vein parameters, the 5×5 convolution kernel possesses higher accuracy than other convolution kernels.

To verify the algorithm’s performance without considering systematic errors, 192 leaf vein images are divided into 6 groups for parameter error measurement without considering the existing systematic error. The results are shown in **Tables 8** and **9**.

**Table 8:** Comparison of measurement results of leaf vein length.

Groups	Scale Bar/(mm∙Pixel^-1^)	Actual Length/mm	Average Pixel/Pixel	Measuring Length/mm	Error/mm
①	0.2645	126.976531	461.62500	122.0998	5.008969
②	0.2645	140.705734	511.15625	135.2008	5.620625
③	0.2645	134.985922	495.28125	131.0019	4.099750
④	0.2645	133.225344	480.5625	127.1088	6.232281
⑤	0.2645	134.341203	490.59375	129.7620	4.579156
⑥	0.2645	150.682344	551.59375	145.8965	4.785797

**Table 9:** Comparison of measurement results of leaf vein width.

Groups	Scale Bar/(mm∙Pixel^-1^)	Actual Width/mm	Average Pixel/Pixel	Measuring Width/mm	Error/mm
①	0.2645	4.266402286	16.81806469	4.448378111	0.186210587
②	0.2645	4.322903885	17.04709003	4.508955313	0.190707262
③	0.2645	4.061104479	15.86600711	4.196558881	0.137903307
④	0.2645	4.236429857	16.8325939	4.452221086	0.223878704
⑤	0.2645	4.284880092	16.78426195	4.439437285	0.154557193
⑥	0.2645	4.210911183	16.47386206	4.357336515	0.146425333

Finally, this study verifies the effectiveness of three image processing methods: Flood Fill Algorithm, Open Operation, and Median Filtering. As shown in **Table 10**, the control variable method is used to evaluate algorithm performance.

**Table 10:** Comparison of parameter measurements for different image processing methods.

Number	F	O	M	*l_acc_* (%)	*W_acc_* (%)
①	–	–	–	40.7995	21.1662
②	✓	–	–	80.7226	72.5739
③	✓	✓	–	86.0089	76.8277
④	✓	✓	✓	96.3642	96.1358

where, F, Flood Fill Algorithm; O, open operation; M, median filtering; *L*_*acc*_ , the accuracy rate of length; *W*_*acc*_ , the accuracy rate of width.

As shown in **Table 10**, the Morphological Skeleton refinement algorithm is directly used to refine leaf veins without any image processing. The algorithm’s accuracy in length is 40.7995% and only 21.1662% in width when measuring leaf vein parameters, which is far from the requirements of actual agriculture. When the Flood Fill Algorithm is added to eliminate leaf vein fine holes, the measurement accuracy of leaf vein length and width is improved to 80.7226% and 72.5739%, respectively. Subsequently, the contour is optimized by using an open operation to eliminate the contour’s fine roughness. Compared with the above method added with the Flood Fill Algorithm, the measurement accuracy of length and width is increased by 5.2863% and 4.2538%, respectively. Finally, the median filter is introduced to remove the noise points of segmentation leaf vein images. Hence, the F-3MS refinement algorithm is formed to extract leaf vein skeleton lines. Its vein length and width measurement accuracy rates are 96.3642% and 96.1358%, respectively. This way, the parameters related to leaf veins can be accurately measured, indicating certain agricultural practical value.

As seen from the analysis in **Table 6**, the leaf vein’s regions can be identified accurately through the MobileNetV2-DeepLabV3+, and the segmentation speed is faster than other networks when extracting plant leaf veins. Therefore, the MobileNetV2-DeepLabV3+ is more suitable for segmenting plant leaf veins. Meanwhile, this proposed method can segment plant veins and measure related parameters in a distributed system. Firstly, the training weight is implanted into the system to segment and extract leaf veins. Subsequently, the segmented veins are further processed using the Convex Hull-Scan method and the F-3MS refinement algorithm. Finally, the related parameters of leaf veins are measured. For example, in the grading of flue-cured tobacco leaves, the extraction of various digital elements in flue-cured tobacco leaves is essential for tobacco grading. To a certain extent, the length, width, and exposure degree of tobacco leaf veins can reflect the growth parts of tobacco leaves. The proposed method in this study can be used to extract digital elements from flue-cured tobacco leaves, which play an essential role in the grading of tobacco leaves.

## 5 Discussion

In deep learning semantic segmentation tasks, the quality of the dataset is one of the core factors affecting the performance of the segmentation network. The result of the model’s training is affected by the small size of the captured image, the different sizes of each image, the different angles of the capture, and the different lighting of the capture. Therefore, the same machine is used for image data acquisition at the same location. Additionally, for the leaf veins’ measurement algorithm to be used in practical agricultural production, the leaves should be kept closer to their original state during image acquisition without any preprocessing operations.

In the semantic segmentation task, the network’s selection, the model structure’s adjustment, and the parameter optimization are all complex. This research focuses mainly on plant leaf veins’ extraction and parameter measurement. Due to the elongated shape of the leaf veins and the need for more texture information, there is no need for very deep network layers for feature extraction. MobileNetV2 is selected as the feature extraction network in this study. Meanwhile, pre-training weights are loaded in the experiments to make the model not be trained from zero. Transfer learning is adopted to train the model and gain a better weight. To shorten the training time, a staged approach to training network models is proposed. The feature extraction network is frozen for training at the first 50 epochs with a mini-batch size of 8, and the entire model is loaded for training after 50 epochs with a mini-batch size of 4.

The advantages and disadvantages of the proposed method and follow-up research are now discussed. Currently, the method of leaf vein segmentation and parameter measurement in this study has the following advantages:

(1) A professional image data acquisition setup is used for image data acquisition and to produce datasets for leaf vein segmentation.
(2) The image data is collected without any filtering, and the samples are closer to the natural field conditions, making the model more generalizable for actual agricultural production.
(3) The MobileNetV2-DeepLabV3+ has shown excellent leaf vein segmentation performance.
(4) The MobileNetV2-DeepLabV3+ can support a 4G graphics card for network training. However, at least conventional segmentation networks often need a 6G graphics card for model training. Hence, most computer configurations can support the training of our network.
(5) The execution of the proposed network is fast, so the execution time for leaf vein segmentation is short, saving computational resources and recognition time.
(6) The Convex Hull-Scan method can repair the leaf veins very well, making it closer to the actual shape.
(7) Due to the fact that the contour of the repaired leaf veins is not smooth, there are many burrs in the skeleton line of the leaf vein extracted by the ordinary refinement algorithm, which leads to a large error in the following parameter measurement. The F-3MS refinement algorithm proposed in this research can eliminate the burr phenomenon and improve the parameter measurement accuracy.

The proposed algorithm also has the following limitations:

(1) This experimental image dataset collected 800 images and later expanded to 3912 images. The dataset samples are still small. The segmentation performance of the network will be improved by adding more image data samples.
(2) The leaf veins are thin strips, and more attention should be paid to the shallow texture information when constructing the network. If multiple shallow feature fusions are used, the segmentation effect of the leaf veins may be better.
(3) Different convolutional network models have different sensitivities to the learning rate. In this research, employing the same learning rate for all convolutional networks may not achieve optimal performance for each network.

Follow-up work is as follows: (1) More images need to be acquired to expand the dataset. (2) Fusion multiple shallow features of the network. (3) To meet the training needs of more computers, the network needs to be compressed further in the next step. (4) For different networks, different parameter tuning methods can be used for optimization, and then the model’s performance is evaluated. (5) This study is used in distributed systems, and the application must be verified in more practical scenarios.

## 6 Conclusions

This study aims to develop a method for automatically measuring leaf vein parameters. It mainly solves problems of slow segmentation speed, partial occlusion of veins, and low measurement accuracy of leaf vein parameters. Experiments show that when using MobileNetV2 as the feature extraction network, MIoU and mPA increased to 81.50% and 92.89%, respectively. Moreover, the segmentation speed is 9.81 frames per second. The model parameters are compressed by 89.375%, down to 5.813M. The measurement accuracy of leaf vein length and width increased to 96.3642% and 96.1358%. Experimental results validate that the proposed algorithm can accomplish the research objective. The study provides a new idea for measuring leaf vein extraction and parameters. Under the studied scenarios, the method can meet the transplant requirements of embedded equipment in agriculture. Future studies will focus on: (1) Transferring the proposed algorithm to other plant leaf vein segmentation and related parameter measurements by fine-tuning the new dataset. (2) Optimizing the algorithm and improving the operating efficiency.

## Data availability statement

The raw data supporting the conclusions of this article will be made available by the authors, without undue reservation.

## Author contributions

BX: methodology, data curation, formal analysis, writing—original draft preparation, writing—review and editing. XL: Conceptualization, methodology, formal analysis. YY: Conceptualization, formal analysis. WG: data curation, formal analysis, writing—review and editing. HW: data curation, writing—review and editing. All authors contributed to the article and approved the submitted version.

## Funding

This research was funded by the National Natural Science Foundation of China (52065033), the Establishment and Application of Artificial Intelligence Grading Model Based on Roasted Tobacco Base Samples in Yunnan Province, China (2021530000241012) and Major Science and Technology Special Project of Yunnan Provincial Science and Technology Department, China (202002AD080001).

## Acknowledgments

We would like to thank the Yunnan Tobacco Quality Supervision and Monitoring Station for providing us with data sources. We also thank the reviewers and the academic editor for their valuable comments and recommendations.

## Conflict of interest

The authors declare that the research was conducted in the absence of any commercial or financial relationships that could be construed as a potential conflict of interest.

## Publisher’s note

All claims expressed in this article are solely those of the authors and do not necessarily represent those of their affiliated organizations, or those of the publisher, the editors and the reviewers. Any product that may be evaluated in this article, or claim that may be made by its manufacturer, is not guaranteed or endorsed by the publisher.

## References

[B1] Barré P. Stöver B. C. Müller K. F. Steinhage V. (2017). LeafNet: A computer vision system for automatic plant species identification. Ecol. Inf. 40, 50–56. doi: 10.1016/j.ecoinf.2017.05.005

[B2] Barth R. Ijsselmuiden J. Hemming J. Henten E. (2018). Data synthesis methods for semantic segmentation in agriculture: A capsicum annuum dataset. Comput. Electron. Agric. 144, 284–296. doi: 10.1016/J.COMPAG.2017.12.001

[B3] Beikmohammadi A. Faez K. Motallebi A. (2022). SWP-LeafNET: A novel multistage approach for plant leaf identification based on deep CNN. Expert Syst. Appl. 202, 117470. doi: 10.1016/j.eswa.2022.117470

[B4] Bosilj P. Aptoula E. Duckett T. Cielniak G. (2020). Transfer learning between crop types for semantic segmentation of crops versus weeds in precision agriculture. J. Field Robot. 37 (1), 7–19. doi: 10.1002/rob.21869

[B5] Bühler J. Rishmawi L. Pflugfelder D. Huber G. Scharr H. Hülskamp M. . (2015). phenoVein – a tool for leaf vein segmentation and analysis. Plant Physiol. 169 (4), 2359–2370. doi: 10.1104/pp.15.00974 26468519 PMC4677892

[B6] Chaturvedi S. S. Gupta K. Prasad P. S. (2020). “Skin lesion analyser: An efficient seven-way multi-class skin cancer classification using MobileNet,” in International conference on advanced machine learning technologies and applications (Singapore: Springer), 1141, 165–176. doi: 10.1007/978-981-15-3383-9_15

[B7] Chen J. Lu Y. Yu Q. Luo X. Adeli E. Wang Y. . (2021). TransUNet: Transformers make strong encoders for medical image segmentation. (United States: arXiv preprint) doi: 10.1109/igarss46834.2022.9883628

[B8] Chen L. C. Papandreou G. Schroff F. Adam H. (2017). Rethinking atrous convolution for semantic image segmentation. (United States: Institute of Electrical and Electronics Engineers) doi: 10.48550/arXiv.1706.05587

[B9] Chen L.-C. Zhu Y. Papandreou G. Schroff F. Adam H. (2018). “Encoder-decoder with atrous separable convolution for semantic image segmentation,” in Proceedings of the European conference on computer vision (ECCV), (Germany: ECCV) Vol. 801-818. doi: 10.1007/978-3-030-01234-2_49

[B10] Cheung W. Hamarneh G. (2009). “N-SIFT: n-dimensional scale invariant feature transform, IEEE Transactions on Image Processing ” in IEEE Transactionson Image Processing, (United States: IEEE Transactions on Image Processing) Vol. 18 (9). doi: 10.1109/TIP.2009.2024578 19502129

[B11] Chollet F. (2017). “Xception: Deep learning with depthwise separable convolutions Conference on Computer Vision and Pattern Recognition (CVPR),” in Proceedings of the IEEE conference on computer vision and pattern recognition (United States: Institute of Electrical and Electronics Engineers (IEEE Xplore)). doi: 10.1109/CVPR.2017.195

[B12] Dalal N. Triggs B. (2005). “Histograms of oriented gradients for human detection,” in 2005 IEEE computer society conference on computer vision and pattern recognition. International Journal of Information System Modeling and Design (IJISMD) (United States) 886–893 (CVPR'05): Ieee). doi: 10.1109/icnc.2013.6818189

[B13] Deepalakshmi P. Prudhvi Krishna T. Siri Chandana S. Lavanya K. Srinivasu P. N. (2021). Plant leaf disease detection using CNN algorithm. Int. J. Inf. Syst. Modeling Design (IJISMD) 12 (1), 1–21. doi: 10.4018/IJISMD.2021010101

[B14] Fid A. Fw B. Pc A. Chen W. C. (2020). ResUNet-a: A deep learning framework for semantic segmentation of remotely sensed data - ScienceDirect. ISPRS J. Photogramm. Remote Sens. 162, 94–114. doi: 10.1016/j.isprsjprs.2020.01.013

[B15] Fuentes-Pacheco J. Torres-Olivares J. Roman-Rangel E. Cervantes S. Juarez-Lopez P. Hermosillo-Valadez J. . (2019). Fig plant segmentation from aerial images using a deep convolutional encoder-decoder network. Remote Sens. 11 (10), 1157. doi: 10.3390/rs11101157

[B16] Ghazi M. M. Yanikoglu B. Aptoula E. (2017). Plant identification using deep neural networks *via* optimization of transfer learning parameters. Neurocomputing 235, 228–235. doi: 10.1016/j.neucom.2017.01.018

[B17] He K. Zhang X. Ren S. Sun J. (2016). “Deep residual learning for image recognition Conference on Computer Vision and Pattern Recognition (CVPR),” in Proceedings of the IEEE conference on computer vision and pattern recognition (United States: Institute of Electrical and Electronics Engineers (IEEE Xplore)). 770–778.

[B18] Howard A. Sandler M. Chu G. Chen L. C. Chen B. Tan M. . (2019). Searching for MobileNetV3. doi: 10.48550/arXiv.1905.02244

[B19] Huang G. Shu Y. Peng S. Li Y. (2022). Leaf photosynthesis is positively correlated with xylem and phloem areas in leaf veins in rice (Oryza sativa) plants. Ann. Bot. 129 (5), 619–631. doi: 10.1093/aob/mcac020 35143609 PMC9007091

[B20] Jiyou Z. Qiang Y. Di Y. Chengyang X. Yang Y. Xiang C. (2019). Extraction and optimization of microscopic image vein network based on eCognition software. Nongye Jixie Xuebao/Transactions Chin. Soc. Agric. Machinery 50 (1), 51–57. doi: 10.1109/icalip.2018.8455845

[B21] Kolhar S. Jagtap J. (2021). Convolutional neural network based encoder-decoder architectures for semantic segmentation of plants. Ecol. Inf. 64, 101373. doi: 10.1016/j.ecoinf.2021.101373

[B22] Konch T. J. Dutta T. Buragohain M. Raidongia K. (2021). Remarkable rate of water evaporation through naked veins of natural tree leaves. ACS omega 6 (31), 20379–20387. doi: 10.1021/acsomega.1c02398.s002 34395986 PMC8359162

[B23] Lee K.-B. Hong K.-S. (2013). An implementation of leaf recognition system using leaf vein and shape. Int. J. Bio-Science Bio-Technology 5 (2), 57–66. doi: 10.3724/sp.j.1087.2009.01707

[B24] Li Y. Chi Z. Feng D. D. (2006). “Leaf vein extraction using independent component analysis,” in 2006 IEEE International Conference on Systems, Man and Cybernetics. IEEE International Conference on Systems, Man and Cybernetics 3890–3894 (United States: IEEE). doi: 10.1109/icsmc.2006.384738

[B25] Li C. Sun C. Wang J. Li F. (2011). Extraction of leaf vein based on improved sobel algorithm and hue information. Trans. Chin. Soc. Agric. Eng. 27 (7), 196–199. doi: 10.1109/iciecs.2009.5364313

[B26] Li Y. Zhang H. Yang T. Ma Z. Li S. (2018). Vein detection method based on fuzzy logic and multiple order morphology. Sci. Silvae Sinicae. 54 (5), 70–77. doi: 10.1109/t2fzz.2013.6613297

[B27] Lu W. Chen J. Xue F. (2022a). Using computer vision to recognize composition of construction waste mixtures: A semantic segmentation approach. Resour. Conserv. Recy. 178, 106022. doi: 10.1016/j.resconrec.2021.106022

[B28] Lu L. Hui L. Ran S. (2022b). Plant leaf segmentation and feature extraction based on multi-view time series images. J. Agric. Mach. 53 (001), 253–260. doi: 10.1360/n112018-00089

[B29] Masuda T. (2021). “Leaf area estimation by semantic segmentation of point cloud of tomato plants,” in Proceedings of the IEEE/CVF International Conference on Computer Vision) (United States: Institute of Electrical and Electronics Engineers (IEEE Xplore)). 1381–1389. doi: 10.1109/iccvw54120.2021.00159

[B30] Ma N. Zhang X. Zheng H.-T. Sun J. (2018). “Shufflenet v2: Practical guidelines for efficient cnn architecture design Computer Vision – ECCV 2018, Part of the Lecture Notes in Computer Science book series (LNIP, volume 11218), ” in Proceedings of the European conference on computer vision (ECCV). (Germany: ECCV) 116–131. doi: 10.1007/978-3-030-01264-9_8

[B31] Ma J. Zhao Z.-L. Lin S. Xie Y. M. (2021). Topology of leaf veins: Experimental observation and computational morphogenesis. J. Mech. Behav. Biomed. Mater. 123, 104788. doi: 10.1016/j.jmbbm.2021.104788 34428694

[B32] Miao C. Xu Z. Rodene E. Yang J. Schnable J. C. (2020). Semantic segmentation of sorghum using hyperspectral data identifies genetic associations. Plant Phenomics 2020:4216373. doi: 10.34133/2020/4216373 33313555 PMC7706332

[B33] Milioto A. Lottes P. Stachniss C. (2018). “Real-time semantic segmentation of crop and weed for precision agriculture robots leveraging background knowledge in CNNs,” in 2018 IEEE international conference on robotics and automation (ICRA). IEEE International Conference on Robotics and Automation (ICRA) 2229–2235 (United States: IEEE). doi: 10.1109/icra.2018.8460962

[B34] Pan L. George-Jaeggli B. Borrell A. Jordan D. Koller F. Al-Salman Y. . (2022). Coordination of stomata and vein patterns with leaf width underpins water-use efficiency in a C4 crop. Plant Cell Environ. 45 (6), 1612–1630. doi: 10.1111/pce.14225 34773276

[B35] Radha R. Jeyalakshmi S. (2014). “An effective algorithm for edges and veins detection in leaf images,” in 2014 World Congress on Computing and Communication Technologies. World Congress on Computing and Communication Technologies (WCCCT) 128–131 (United States: IEEE). doi: 10.1109/wccct.2014.1

[B36] Rodríguez-Ramírez E. C. García-Morales L. J. Alcántara-Ayala O. Vázquez-García J. A. Luna-Vega I. (2021). Leaf vein morphological variation in four endangered Neotropical magnolia species along an elevation gradient in the Mexican tropical montane cloud forests. Plants 10 (12), 2595. doi: 10.3390/plants10122595 34961066 PMC8703730

[B37] Ronneberger O. Fischer P. Brox T. (2015). “U-Net: Convolutional networks for biomedical image segmentation, ” in International Conference on Medical Image Computing and Computer-Assisted Intervention Computer Vision and Pattern Recognition (United States: arXiv preprin). doi: 10.1007/978-3-319-24574-4_28

[B38] Samanta G. Chakrabarti A. Bhattacharya B. B. (2018). “Extraction of leaf-vein parameters and classification of plants using machine learning,” in Proceedings of International Conference on Frontiers in Computing and Systems. Part of the Advances in Intelligent Systems and Computing book series (AISC, volume 1255) 579–586 (Singapore: Springer). doi: 10.1007/978-981-15-7834-2_54

[B39] Sandler M. Howard A. Zhu M. Zhmoginov A. Chen L.-C. (2018). “Mobilenetv2: Inverted residuals and linear bottlenecks,” in Proceedings of the IEEE conference on computer vision and pattern recognition. Conference on Computer Vision and Pattern Recognition (CVPR) (United States: Institute of Electrical and Electronics Engineers (IEEE Xplore)) 4510–4520. doi: 10.1109/cvpr.2018.00474

[B40] Selda J. D. S. Ellera R. M. R. Cajayon L. C. Linsangan N. B. (2017). “Plant identification by image processing of leaf veins,” in Proceedings of the International Conference on Imaging, Signal Processing and Communication International Conference on ent Computing and Control Systems (ICICCS). (United States: Institute of Electrical and Electronics Engineers (IEEE Xplore)) 40–44. doi: 10.1145/3132300.3132315

[B41] Sun K. Xiao B. Liu D. Wang J. (2019). “Deep high-resolution representation learning for human pose estimation,” in 2019 IEEE/CVF conference on computer vision and pattern recognition (CVPR), (United States: Institute of Electrical and Electronics Engineers) 5686–5696. doi: 10.1109/CVPR.2019.00584

[B42] Too E. C. Yujian L. Njuki S. Yingchun L. (2019). A comparative study of fine-tuning deep learning models for plant disease identification. Comput. Electron. Agric. 161, 272–279. doi: 10.1016/j.compag.2018.03.032

[B43] Ulku I. Akagündüz E. (2022). A survey on deep learning-based architectures for semantic segmentation on 2d images. Appl. Artif. Intell. 36 (1), 1–45. doi: 10.1080/08839514.2022.2032924

[B44] Wang W. Li Y. Zou T. Wang X. You J. Luo Y. (2020). A novel image classification approach *via* dense-MobileNet models. Mobile Inf. Syst. 2020, 7602384 doi: 10.1155/2020/7602384

[B45] Wen W. Li B. Li B.-J. Guo X. (2018). A leaf modeling and multi-scale remeshing method for visual computation via hierarchical parametric vein and margin representation. Front. Plant Sci. 9. doi: 10.3389/fpls.2018.00783 PMC6029520 29997632

[B46] Westphal E. Seitz H. (2021). A machine learning method for defect detection and visualization in selective laser sintering based on convolutional neural networks. Addit. Manuf. 41, 101965. doi: 10.1016/j.addma.2021.101965

[B47] Wu T. Tang S. Zhang R. Cao J. Zhang Y. (2021). CGNet: A light-weight context guided network for semantic segmentation. IEEE Trans. Image Process. 30, 1169–1179. doi: 10.1109/TIP.2020.3042065 33306466

[B48] Ye M. Wu M. Zhang H. Zhang Z. Zhang Z. (2021). High leaf vein density promotes leaf gas exchange by enhancing leaf hydraulic conductance in oryza sativa l. plants. Front. Plant Sci. 12. doi: 10.3389/fpls.2021.693815 PMC8573028 34759936

[B49] Zhang P. Dong T. Jin H. Pei D. Pervaiz T. Ren Y. . (2022). Analysis of photosynthetic ability and related physiological traits in nodal leaves of grape. Sci. Hortic. 304, 111251. doi: 10.2139/ssrn.3998731

[B50] Zhang X. Zhou X. Lin M. Sun J. (2018). “Shufflenet: An extremely efficient convolutional neural network for mobile devices,” in Proceedings of the IEEE conference on computer vision and pattern recognition. Conference on Computer Vision and Pattern Recognition (CVPR) (United States: Institute of Electrical and Electronics Engineers (IEEE Xplore)) 6848–6856. doi: 10.48550/arXiv.1707.01083

[B51] Zhao G. Pietikainen M. (2007). Dynamic texture recognition using local binary patterns with an application to facial expressions. IEEE Trans. Pattern Anal. Mach. Intell. 29, 915–928. doi: 10.1109/TPAMI.2007.1110 17431293

[B52] Zhao H. Shi J. Qi X. Wang X. Jia J. (2016). Pyramid scene parsing network (United States: IEEE Computer Society). doi: 10.1109/CVPR.2017.660

[B53] Zheng X. Wang X. (2010a). “Leaf vein extraction using a combined operation of mathematical morphology,” in International Conference on Information Engineering & Computer Science. International Conference on Information Engineering and Computer Science (ICIECS) (United States: Institute of Electrical and Electronics Engineers (IEEE Xplore)) doi: 10.1109/iciecs.2010.5677786

[B54] Zheng X. Wang X. (2010b). Leaf vein extraction based on grayscale morphology. Int. J. Image Graphics Signal Process. 2 (2), 25. doi: 10.5815/ijigsp.2010.02.04

[B55] Zhu J. Yao J. Yu Q. He W. Xu C. Qin G. . (2020). A fast and automatic method for leaf vein network extraction and vein density measurement based on object-oriented classification. Front. Plant Sci. 11. doi: 10.3389/fpls.2020.00499 PMC7214732 32431721

[B56] Zifen H. Junxuan H. Qiang L. Yinhui Z. (2021). Apple leaf disease segmentation based on asymmetric mixed wash convolutional neural network. J. Agric. Mach. 52 (8), 10. doi: 10.6041/j.issn.1000-1298.2021.08.022

